# Interpreting patient-reported outcomes after ischemic stroke: defining minimal important difference in EQ-5D across recovery phases

**DOI:** 10.1186/s12955-026-02524-w

**Published:** 2026-03-21

**Authors:** Pingping Li, Min Zhao, Yining Huang, Weiping Sun, Luying Wang, Xuejing Jin, Feng Xie, Hongchao Li

**Affiliations:** 1 School of International Pharmaceutical Business, Economics & Arts Building, Jiangning Campus, China Pharmaceutical University, 639 Longmian Avenue, Jiangning District, Nanjing City, Jiangsu Province 211198 China; 2 Department of Neurology, Peking University First Hospital, Beijing, 100871 China; 3 Center for Pharmacoeconomics and Outcomes Research, China Pharmaceutical University, Nanjing, 210009 China; 4 Centre for Evidence-based Chinese Medicine, Beijing University of Chinese Medicine, No. 11, Bei San Huan Dong Lu, Chaoyang, Beijing, 100029 China; 5 Department of Health Research Methods, Evidence, and Impact, Center for Health Economics and Policy Analysis, Faculty of Health Sciences, McMaster University, 1280 Main Street West, CRL223, Hamilton, ON L8S 4K1 Canada

**Keywords:** EQ-5D, Minimal important difference, Stroke, Recovery phase, Longitudinal study

## Abstract

**Background:**

The EQ‑5D is widely applied to measure patient-reported outcomes, yet its minimally important difference (MID) has not been clearly established across distinct recovery phases after stroke. This study aimed to determine phase‑specific MIDs in EQ‑5D following stroke and to explore heterogeneity by estimation method, direction of change, and stroke etiology.

**Methods:**

A total of 9978 adults with neuroimaging‑confirmed acute ischemic stroke were included in a prospective longitudinal cohort study. EQ‑5D and modified Rankin Scale (mRS) scores were recorded at admission (V1), hospital discharge (V2), 3-month (V3), and 1-year since admission (V4). Anchor-based MIDs were estimated at both group and individual levels and triangulated by distribution-based and instrument-defined approaches. Changes during the recovery phases (V1–V2, V2–V3, and V3–V4) were grouped into 3 categories: improved, no change, and deteriorated. Subgroup analyses were conducted according to the TOAST classification. Credibility of MID estimates was assessed using a validated instrument for anchor-based methods.

**Results:**

Phase-specific group-level MIDs for improvement decreased over time: anchor-based estimates were 0.19 at V2, 0.14 at V3, and 0.11 at V4, while deterioration MIDs were smaller. Distribution-based and instrument-defined estimates fluctuated slightly around the anchor-based values but followed a similar downward trend over time. Individual‑level analyses yielded MIDs with acceptable discriminative power (area under the curve ≥ 0.70) only for improvement at V2 (0.10) and V3 (0.01). Cardioembolic strokes had higher MIDs than large-artery atherosclerosis and small-artery occlusion, while baseline utilities showed the reverse. Credibility assessment confirmed high reliability.

**Conclusion:**

This study provides the phase‑specific MIDs for utility measures after ischemic stroke, showing a declining trend from acute to chronic recovery and confirming robustness across multiple estimation methods. While group‑level MID ranges are recommended for effect size interpretation, trial design, and evidence certainty rating, individual‑level thresholds should be reserved for personalized evaluation. These values assist in the interpretation of patient‑reported outcome changes and evaluation of healthcare interventions across different recovery phases.

**Trial registration:**

ClinicalTrials.gov NCT02470624. Registered 10 June 2015.

**Supplementary Information:**

The online version contains supplementary material available at 10.1186/s12955-026-02524-w.

## Background

Stroke remains the second leading cause of death and the third leading cause of disability globally [1]. Ischemic stroke (IS) accounts for approximately 85% of all strokes and is associated with substantial mortality and economic burden [2]. In China, the incidence and recurrence of stroke continue to rise over recent decades [3–5]. Hospitalization cost for IS rose nearly 40% from 2010 to 2020 [6], and rehabilitation now exceeds ¥63,000 per patient with limited insurance coverage [7]. Stroke recovery has long been evaluated using clinician-reported instruments such as the National Institutes of Health Stroke Scale (NIHSS), Barthel Index (BI), and the modified Rankin Scale (mRS) [8]. While these instruments effectively capture neurological deficits and functional status, they may not fully reflect patients’ own perceptions of recovery. The growing emphasis on patient-reported outcomes (PROs) has highlighted health utility measures such as the EQ-5D, which are crucial for evaluating quality of life and health technology assessment [9–11]. Interpreting the clinical relevance and importance of observed changes for EQ-5D requires a threshold for what constitutes a meaningful change. The minimally important difference (MID), defined as the smallest score change that patients perceive as clinically meaningful, is the most widely used method for this purpose [12].

Although MIDs for EQ-5D have been established in various chronic conditions and have revealed the association with treatment type and baseline score, evidence in stroke remains limited [13–15]. Existing studies are often constrained by small sample sizes, reliance on single anchors or one distribution-based index [16–18]. Furthermore, no previous study has investigated how MIDs vary across distinct recovery phases or stroke etiological subtypes classified by the Trial of Org 10,172 in Acute Stroke Treatment (TOAST). Given that health utility preferences are population-specific, MIDs for utility also depend on local population value sets [13, 19, 20].

Therefore, this study aimed to estimate phase-specific MIDs for EQ-5D-3-L in patients after acute ischemic stroke (AIS) using real-world, longitudinal registry data. Secondary objectives were to compare MID estimates across three commonly used methods, namely, anchor-based, distribution-based, and instrument-defined approaches; to evaluate MIDs by TOAST subtypes; and to assess MID using a credibility instrument specifically designed for anchor-based MID [21].

### Methods

### Study design and participants

We used the data from the Chinese Acute Ischemic Stroke Treatment Outcome Registry (CASTOR; ClinicalTrials.gov Identifier: NCT02470624). CASTOR is a prospective, longitudinal, multicenter observational registry designed to evaluate outcomes among patients with AIS. Between March 2015 and December 2018, 40 tertiary hospitals across 19 provinces in China enrolled eligible patients. The protocol was approved by the institutional review board of the Peking University First Hospital (Approval No. 2015[922]) and corresponding ethics committees at each participating center before study initiation. All participants provided written informed consent prior to enrollment. Eligibility criteria (detailed in the published protocol [22]) required patients to be adults (≥18 years) with neuroimaging-confirmed AIS.

### Measures and scales used

Health-related quality of life (HRQOL) was assessed in this study using the EQ-5D-3-L and functional outcome using the mRS at admission (V1), hospital discharge (V2), 90 ± 14 days since admission (V3), and 360 ± 28 days since admission (V4). In the present analysis, MIDs for EQ-5D-3-L health utility were calculated separately for three study periods: V1–V2, V2–V3, and V3–V4. These phases were selected because they correspond to the acute, subacute and chronic phases of IS, which enhances clinical relevance.

The EQ-5D-3-L, a validated multi-attribute utility instrument to assess HRQOL, consists of two core components: a descriptive system measuring health state and a visual analogue scale (EQ-VAS) [23]. The descriptive system includes five dimensions: mobility, self-care, usual activities, pain/discomfort, and anxiety/depression. Each dimension has 3 response categories (1 = no problems, 2 = some problems, 3 = extreme problems). This instrument has been validated in the stroke population, showing satisfactory test-retest reliability (intraclass correlation coefficient [ICC] = 0.75–0.81), adequate construct validity, and superior responsiveness to clinical improvements compared with the EQ-5D-5-L index and EQ-VAS. [24, 25] Two EQ-5D-3-L value sets have been published for China [26, 27]. The 2014 version was used to calculate health utility in this study [27]. The EQ-5D-3-L was the target instrument for estimating MIDs, as it allows quantification of patients’ perceived changes in health status into utility values relevant for both clinical and economic evaluations.

In stroke research, MIDs have been established for the BI and mRS, in addition to the NIHSS [18]. As mRS was collected in the CASTOR study, it was selected as the anchor in the present analysis. The mRS is a clinician-reported outcome tool designed to evaluate global disability in stroke. It categorizes functional status into seven hierarchical grades (0–6), where 0 denotes no symptoms and 6 indicates death. The mRS has shown good reliability, validity, and responsiveness [8]. Large-scale prospective studies have shown that a ≥ 1-point change reflects meaningful functional progression [28], a finding further corroborated by a 2017 Delphi consensus of 122 academic stroke neurologists [29], which established any single-grade shift to represent the MID in stroke populations

### Statistical analysis

To ensure data quality and sample integrity, if the proportion of missing data for EQ-5D-3-L health state items and mRS levels is less than 5% (as in this study), cases with missing values were directly excluded; if the missing rate falls between 5 and 30%, multiple imputation was applied. All analyses were performed in the R statistical programming language.

#### Interpretation of minimal important difference

The mRS served as the anchor for determining the MID of EQ-5D-3-L in this study. The health state level transitions of mRS were classified as: (1) no change (non-responders), (2) minimal improvement or deterioration (responders, defined as a change of −1 or + 1 grade), (3) major improvement or deterioration (more than −1 or +1; encompassing both moderate and large effects) [29]. Patients with either minimal change or no change were included in the anchor-based analysis. Four anchor-based statistical methods were employed to calculate the MID [30]: (1) average change (AC), defined as the mean EQ-5D utility change of responders (within-group difference); (2) change difference (CD), the difference in the average change score of respondents and non-responders (between-group difference); (3) linear regression of EQ-5D change on mRS change, with the regression coefficient of the mRS shift interpreted as the MID; (4) receiver operating characteristic (ROC) curve analysis, which required an area under the curve (AUC) >0.7 for acceptable discriminative ability, using EQ-5D score change as the test measure and mRS score change as the reference, with the threshold defined by the maximum Youden’s index. The ROC-based MID represents an individual-level threshold, whereas the other three methods provide group-level estimates.

Instrument-defined method calculates the average health utility difference between baseline health state and single-level transitions to adjacent states, taking the specific clinical profile of IS into account [19]. Participants with baseline profiles of 11,111 were excluded, as no better health state was possible.

All patients, encompassing those with minor, major, or no change, were included in the distribution-based analysis. We used four metrics: 0.2SD, 0.5SD, standard error of measurement (SEM), and 0.5 effect size (ES). Consistent with established HRQoL research, 0.2SD represent a minimal change [31], while the other three metrics are widely regarded as robust indicators of minimal change [32]. Collectively, these four parameters are among the most frequently utilized metrics for MID estimation in HRQoL studies [30]. In addition, we calculated the minimal detectable change (MDC) which represents a statistical threshold for the smallest change detectable beyond measurement error [30, 33]. We used the MDC to ascertain whether our MID estimates exceeded measurement error. Specifically, we calculated the ratio of MID to MDC at two confidence levels (90 and 95%), and at both individual and group levels. Ratios greater than one indicated that the MID exceeded measurement errors [34]. Detailed calculation methods for all distribution‑based indices are provided in Supplementary Material A.

Credibility refers to how well the study design and implementation minimize the risk of inaccurate MID estimates. Devji et al. (2020) developed a credibility assessment tool for anchor-based MID studies [21], which, along with its recent extension [35], provides a framework for evaluating methodological rigor. The tool includes five core criteria and four additional criteria for transition rating anchors. The overall credibility was rated as high or low, based on the core criteria, with a high rating requiring at least three’definitely yes’ or’to a great extent’ responses, and a low rating if less than two core criteria met this standard.

#### Subgroup analysis

TOAST classification is internationally recognized as the preferred standard for etiological diagnosis of IS [2]. This criterion categorizes the causes of AIS patients into five types: large-artery atherosclerosis (LAA), cardioembolic infarction (CE), small-artery occlusion (SA), stroke of other explicit etiology (SOE), and stroke of undetermined etiology (SUE). Subtype-level analyses were conducted because pathophysiology, baseline severity, and recovery trajectories vary across subtypes. The Kruskal-Wallis (K-W) test was used to compare whether baseline and follow-up scores of EQ-5D differed significantly across TOAST subgroups. For outcomes with significant post-hoc differences (*p* < 0.05), subgroup-specific MIDs were calculated.

## Results

### Sample characteristics

**Fig. 1:**
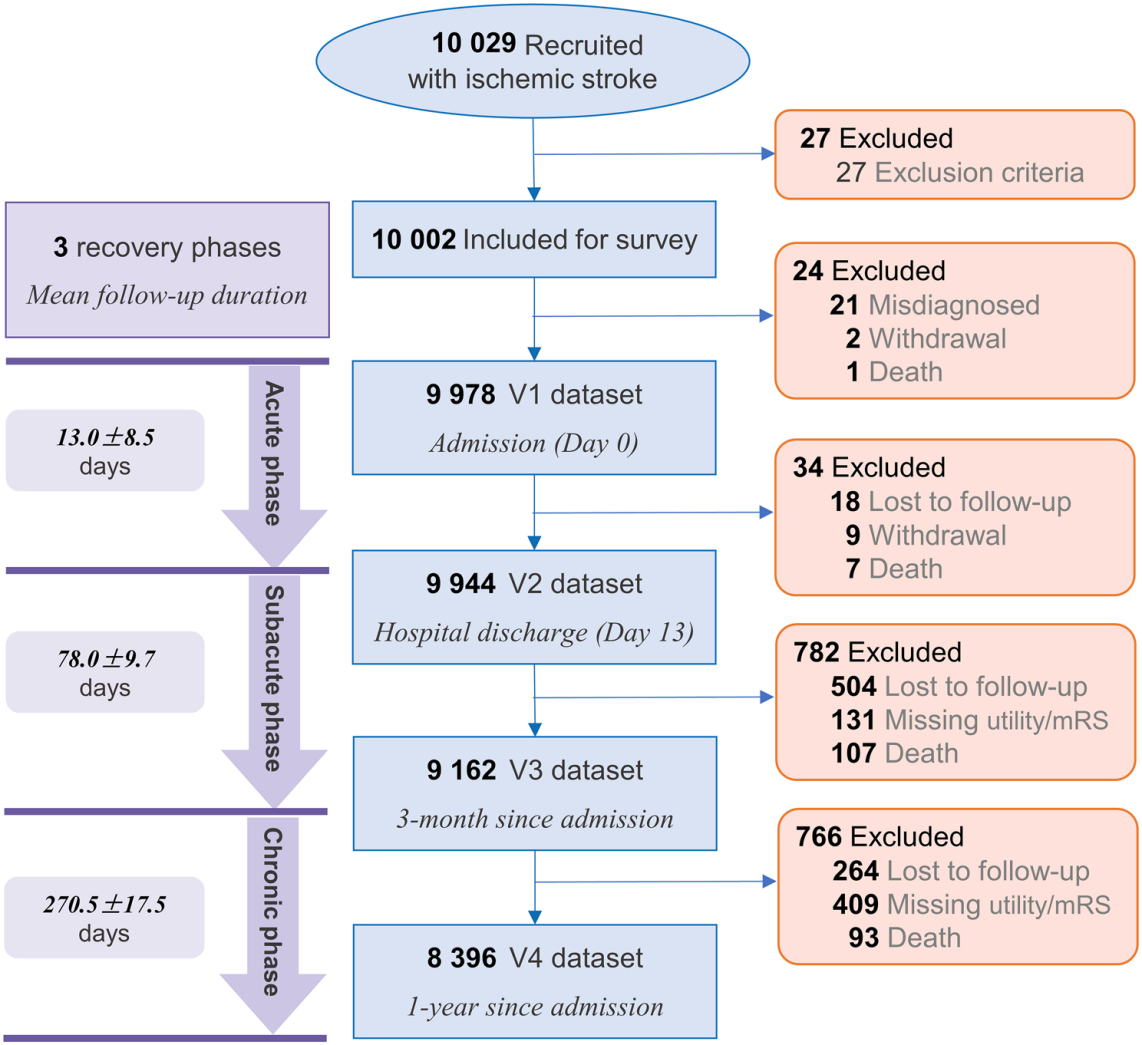
Flow chart of patients

From 10,029 enrolled patients, 10,002 were eligible. The analytical cohorts included 9978 (99.8%), 9944 (99.4%), 9162 (91.6%), and 8396 (83.9%) patients at V1 to V4, respectively (Fig. 1). Table 1 presents the baseline characteristics of the study population. Detailed characteristics for this cohort have been previously reported by Wang et al. [36]. At admission, 47.1% of the Chinese patient cohort presented with mild stroke severity (NIHSS score 1–4) [36].

**Table 1:** Baseline patient characteristics

	Participants, No. (%)		
Characteristic	V1 (N = 9978)	V2 (N = 9944)	V3 (N = 9162)
Age, mean (SD), y	64.0 (11.9)	64.0 (11.9)	63.9 (11.9)
Male sex	6564 (65.8)	6564 (65.8)	6564 (65.8)
BMI, mean (SD)^a^	24.6 (3.40)	24.56 (3.42)	24.56 (3.42)
TOAST classifications^b^			
LAA (n)	2890 (64.6)	2880 (64.6)	2731 (64.2)
CE (n)	190 (4.2)	189 (4.2)	167 (3.9)
SA (n)	1131 (25.3)	1129 (25.3)	1107 (26.0)
Other determined cause (n)	105 (2.3)	105 (2.4)	100 (2.3)
Undetermined cause (n)	159 (3.6)	158 (3.5)	151 (3.5)
NIHSS Scores, mean (SD)	5.3 (4.98)	3.6(4.24)	NA
Treatments specific for AIS			
IV thrombolysis	859 (8.6)	859 (8.6)	801 (8.7)
Antiplatelet	9536 (95.7)	9511 (95.6)	8764 (95.7)
Anticoagulant	1460 (14.6)	1455 (14.6)	1318 (14.4)
Volume expanders	1384 (13.9)	1384 (13.9)	1316 (14.4)
Neuroprotective agents	9380 (94.1)	9355 (94.1)	8614 (94.0)
Cerebral perfusion-enhancing agents	8718 (87.5)	8694 (87.4)	7999 (87.3)
TCM	7591 (76.1)	7578 (76.2)	6992 (76.3)
Others	348 (3.5)	348 (3.5)	321 (3.5)

Abbreviations: TOAST, Trial of Org 10,172 in Acute Stroke Treatment; LAA, large-artery atherosclerosis; CE, cardioembolic stroke; SA, small-artery occlusion; NIHSS, National Institution of Health Stroke Scale; NA, not applicable; AIS, acute ischemic stroke; IV, intravenous injection; TCM, traditional Chinese medicine

^a^ BMI data available for 2487, 2481, 2318 patients for V1, V2, and V3 respectively

^b^ TOAST classifications data available for 4475, 4461, and 4256 patients for V1, V2, and V3 respectively

*Note.* V1, hospital admission; V2, hospital discharge; V3, 90 ± 14 d post-admission; V4, 360 ± 28 d post-admission

### Longitudinal HRQOL Trends

Significant improvements were observed in health utility (greatest from V1–V2; 1-year AUC: 0.802 [95% CI, 0.797 to 0.807]) and EQ-VAS (1-year AUC: 83.33 [95%CI, 83.02 to 83.64]) (both *p* < 0.001). Concurrently, moderate-to-severe disability (mRS 3–5) declined substantially from 43% at V1 to 15% at V4 (See eTable 1). Based on mRS-defined meaningful functional progress (See eFig. 1), the proportion of patients with functional improvement from V1 rose across visits: 47% at V1–V2, 61% at V1–V3, and 68% at V1–V4, while the proportion with unchanged status declined correspondingly from 46% to 31 and 25%. When analysed across three consecutive recovery phases, the proportion showing meaningful improvement decreased stepwise (V1–V2: 47%; V2–V3: 34%; V3–V4: 25%), whereas the proportion with unchanged status increased in parallel (46, 57, and 65%, respectively). Only a small proportion of patients (7–9%) demonstrated meaningful deterioration at any time.

### MID

EQ-5D-3-L correlated strongly with mRS across visits (all *r* > 0.75, *p* < 0.001; See eFig. 2, left panel). For score changes, correlations were moderate to high (all *r* > 0.33, *p* < 0.001; See eFig. 2, right panel). Although score changes were skewed (See eFig. 3, *p* < 0.001), the large sample size justified using the mean rather than the median to estimate the MID. Within each timepoint (V2, V3, V4), patients with minimal improvement had greater mean utility score changes than those with no meaningful change or minimal deterioration (*p* < 0.001; Table 2, horizontal comparison). When compared across timepoints, significant differences were observed between the minimal improvement and no meaningful change groups, but not for the minimal deterioration group (*p* < 0.001; Table 2, vertical comparison).

**Table 2:** AC‑driven MIDs and proportion of patients above thresholds by recovery phases

Analysis type	Timepoints	Minimal improvement^a^	No change ^a^	Minimal deterioration ^a^	ANCOVA *P* value
Within-timepoint	V2 from V1	0.22	0.08	−0.09	<0.0001
		3477 (35.0%)	5685 (57.2%)	782 (7.8%)	
	V3 from V2	0.15	0.04	−0.08	<0.0001
		2454 (26.8%)	5660 (61.8%)	1048 (11.4%)	
	V4 from V3	0.12	0.02	−0.09	<0.0001
		1917 (22.8%)	5544 (66.0%)	935 (11.1%)	
Across-timepoint	V2 vs V3 vs V4	<0.001	<0.001	0.954	

Abbreviation: AC, average change; MID, minimally important difference; ANCOVA, analysis of covariance

^a^ Minimal improvement: 1‑point decrease in mRS; no change: 0‑point change in mRS; minimal deterioration: 1‑point increase in mRS

*Note.* V1, hospital admission; V2, hospital discharge; V3, 90 ± 14 d post-admission; V4, 360 ± 28 d post-admission. AC‑driven MID was defined as the mean change in EQ‑5D utility among responders. *n* (%) indicates the number (percentage) of patients whose utility change exceeded this mean change

**Fig. 2:**
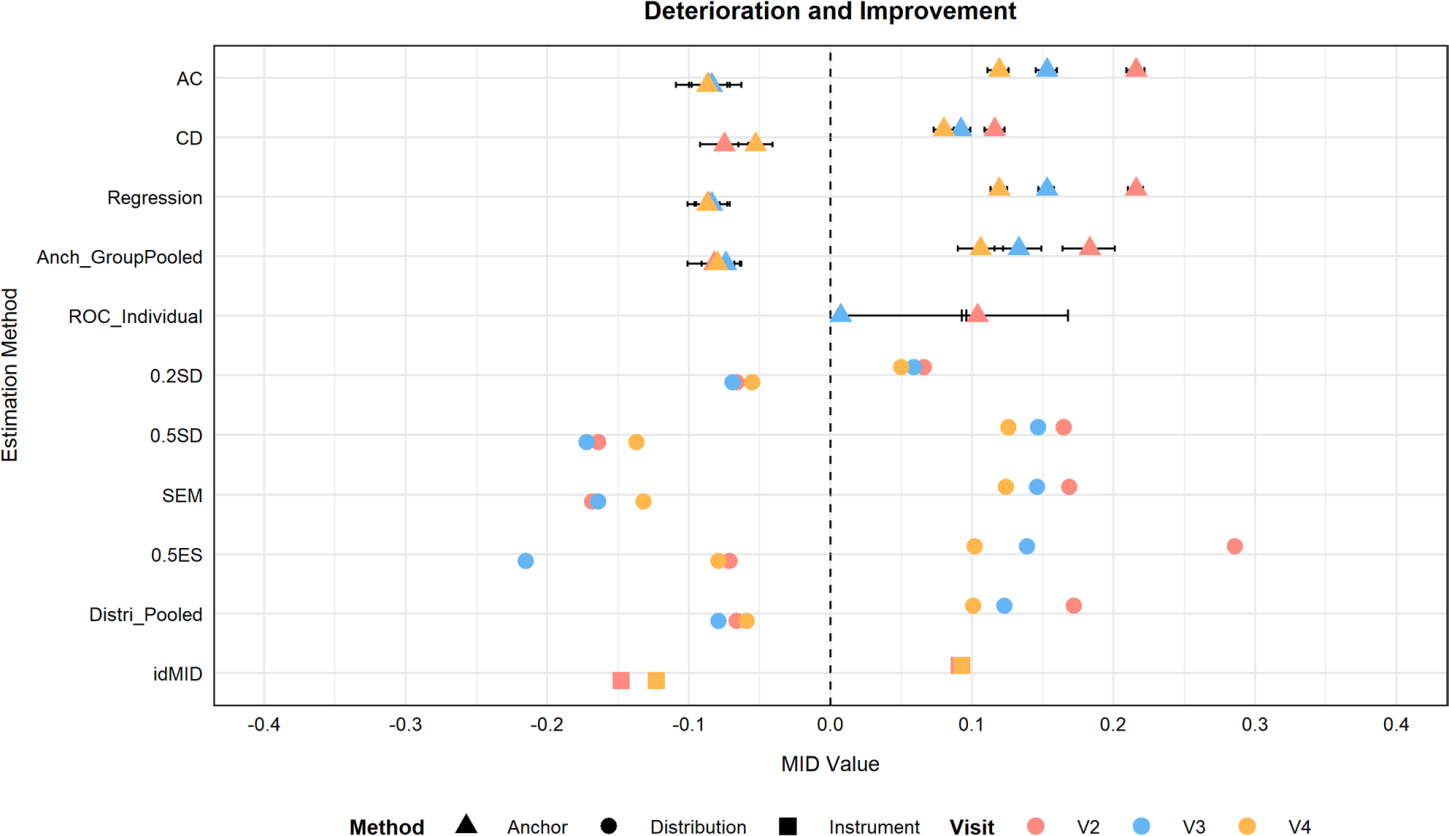
Distribution of minimal importance differences (MIDs) across three recovery phases. Abbreviations: AC, average change; CD, change difference; Anch_GroupPooled, pooled group-level estimates from anchor-based methods; ROC, receiver operating characteristic; SD, standard deviation; SEM, standard error of measurement; ES, effect size; Distri_Pooled, pooled estimates from distribution-based methods; idMID, instrument-defined minimally important difference

See eTable 2 and Figure 2 summarise MID estimates across phases, with anchor‑based results as primary. The mean change for improvement in responders declined over time: acute phase 0.22 (95% CI, 0.21 to 0.22), subacute 0.15 (95% CI, 0.15 to 0.16), and chronic 0.12 (95% CI, 0.11 to 0.13). In contrast, for patients who experienced minimal clinically important deterioration, the MIDs did not differ significantly across the three recovery phases. Regression-derived MIDs approximated the mean change results. Linear regression diagnostics supported the model validity (See eFigures 4–5). Table 2 also shows the number of patients exceeding the AC‑driven MID. The proportion of patients exceeding the improvement threshold decreased across phases, with most showing no meaningful change and few deteriorating. To triangulate anchor‑based estimates, MIDs were also derived using distribution‑based and instrument‑defined approaches (Fig. 2). Distribution‑based values (ranged 0.05–0.29) closely matched anchor‑based estimates (0.10–0.22). Instrument‑defined analyses yielded differences of 0.11 and 0.12 (See eFigure 6).

**Fig. 3:**
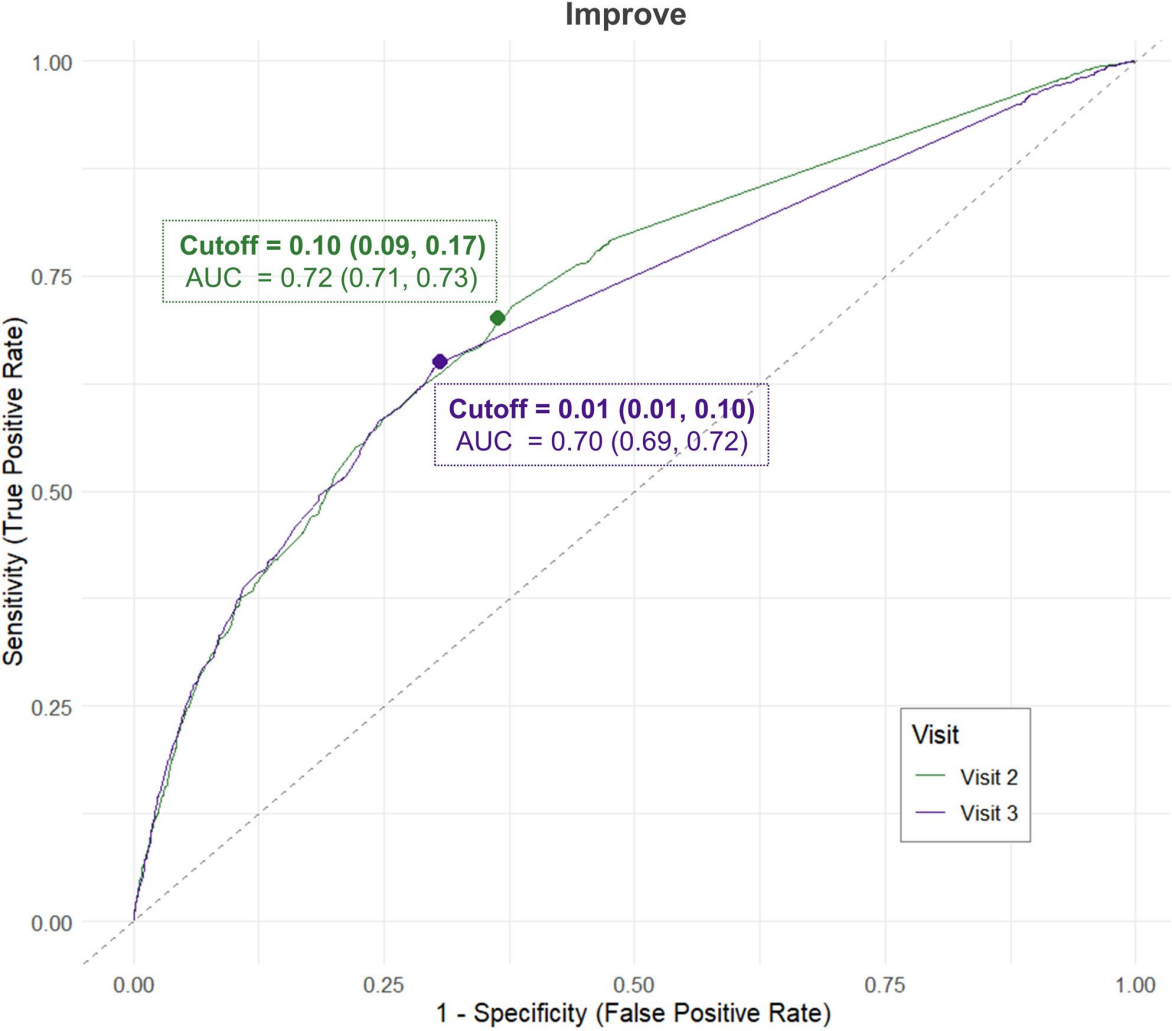
Receiver operating characteristic (ROC) curves for improvement

ROC analyses provide individual‑level thresholds. However, because of the small number of patients with deterioration and the resulting imbalance between positive (deterioration) and negative (no change) cases (e.g., V2: 430 vs 4570; See eTable 3), ROC estimates were obtained only for the improvement direction at V2 and V3 (AUC 0.70–0.72; Fig. 3). These optimal cut‑points were 0.10 at V2 and 0.01 at V3.

All group-level anchor-based MIDs were greater than the MDC_95_ and MDC_90_ (See eFigure 7), indicating that these values reflect changes detectable beyond measurement error. In contrast, the ROC‑derived MIDs were lower than the corresponding individual‑level MDC values. Sensitivity, specificity, and predictive values for both improvement and deterioration, together with the individual‑level MDCs, are showed in See eTable 4. The result of credibility assessment showed 4 out of 5 criteria fully met, confirming high trustworthiness of the established MIDs (rank of 2 out of 11; higher rank means higher credibility; See eTable 5).

### Subtype-specific MIDs

Baseline health utility and the corresponding MIDs significantly varied across TOAST subtypes (*p* < 0.001). Specifically, the utility levels followed the order of SA > LAA >CE across visits 1–4, while comparisons involving SUE and SOE were mostly non‑significant (*p* > 0.05; See eTables 6–7 and eFigure 8). Based on the high heterogeneity within the SUE and SOE subtypes, SA, CE, and LAA were selected as subgroups for subtype‑specific MID estimation. Across all visits and methods, MIDs consistently exhibited an reverse relationship with baseline utility values, following the order of CE > LAA >SA (See eFigure 9). For example, MID estimates at V2 across all change directions and methods followed a descending order: CE subtype (range: 0.073–0.270; 4% of available TOAST sample) >LAA (0.065–0.227; 65%) ≈ overall population (0.065–0.249) >SA subgroup (0.048–0.222; 25%).

## Discussion

This study establishes, for the first time, phase-specific MIDs for the EQ-5D-3-L after AIS. MIDs demonstrated a consistent decline from acute to chronic phases, showing an inverse relationship with baseline utility values. Subgroup analyses also revealed the increasing MIDs with decreasing baseline score among different TOAST classification. These findings collectively indicate that patients with lower baseline health status required a greater magnitude of change to perceive a clinically meaningful improvement; this highlights the essential role of baseline utility in interpreting MIDs for both clinical and research applications.

Consistent with prior MID research, our estimates demonstrated variability across directions of change [37, 38]. Using anchor-based and distribution‑based methods, MID estimates for deterioration were smaller than those for improvement or overall changes (See eTable 2). Existing reviews of EQ-5D MID have generally reported smaller thresholds for deterioration than improvement [13, 39, 40], although some studies have identified the opposite [14, 37, 41]. These differences may be attributable to patient sensitivity to health decline or variations among disease categories. The smaller MID for deterioration observed in this study suggests an asymmetric responsiveness, where patients are more sensitive to health decline than to improvement [42]. This aligns with the concept of loss aversion, where a smaller threshold is required for patients to perceive a meaningful deterioration in their health status [43].

Anchor-based MID estimation relies on two primary statistical approaches: the AC and the CD methods. The AC method quantifies within-group differences and may overestimate change due to natural recovery and other non-specific effects, thus representing a minimal important change (MIC) [42]. In contrast, the CD method captures between-group differences by accounting for both responders and non-responders, providing a more conservative MID. In our study, AC-based values were consistently higher than CD-based values across all time points and directions of change, echoing the findings of Qin et al. [42]. The gap between AC and CD estimates narrowed over time, reflecting increasing utility gains in patients with unchanged mRS scores.

Subgroup analyses revealed that MIDs were inversely related to baseline health utility values across TOAST subtypes, together with two EQ-5D MID reviews [13, 15]. Specifically, patients with CE stroke typically present with greater neurological deficits and lower baseline utility. These patients exhibited higher MID thresholds compared to those with SA or LAA subtypes, suggesting that more severely affected individuals may require more substantial functional gains to perceive a meaningful recovery.

Compared to previous MID of 0.10 at 3–4 weeks reported in a Taiwanese study [16], our estimate (CD: 0.14) at V2 (acute phase: median 13.0 ± 8.5 days) was slightly larger, likely due to our population’s lower baseline utility (0.55 vs. 0.72), different value sets and anchor choice. Similarly, our MID for improvement over the 9-month interval (V3 to V4, 0.10) exceeded the 0.08 reported in a Korean study over a comparable 10-month period, with explanations including value set and our cohort’s higher proportion of patients with no baseline disability (36.2% vs. 12.5%); we also calculated a MID of 0.10 for the V1–V4 interval (11 months), which was approximately equal to that for V3–V4, although our primary analysis focused on the more clinically relevant comparison betweenV4 and V3. Our pooled anchor-based MIDs across all recovery phases (0.08–0.17) fell within the interquartile range (0.05–0.17) reported in a systematic review of EQ-5D-3-L utility MIDs [13]. The distribution-based (0.10–0.16) and instrument-defined estimates (0.11–0.12) in our study exceeded the review’s median pooled distribution-based value (0.08) and the upper range (0.08) of instrument-defined MIDs reported across four countries, likely reflecting our larger sample size and the use of a different value set, respectively. Furthermore, MID values in this study were pooled using mean values, in line with common practice. It should be noted, however, that methodologies for integrating MID estimates or selecting an optimal value within a single study remain undefined [43, 44], and future research could seek to identify pooled MID guided by clinical expertise.

### Strengths and limitations

Strengths of this study include its large sample size and the phase‑specific assessment across acute, subacute, and chronic stages after AIS. This design better captures the heterogeneity of recovery potential and treatment strategies, with acute phase management focused on life‑saving and clinical stabilization through urgent time‑window interventions; the subacute phase emphasizing neurological recovery and rehabilitation training, supported by neurotrophic or neuroprotective agents; and the chronic phase prioritizing functional maintenance and secondary prevention of recurrence, alongside attention to psychological and social adaptation. In addition, we employed multiple methods to triangulate MID estimates and further tested their robustness and validity using bootstrap resampling, a credibility instrument, and MDC.

Several limitations should be noted. First, we used EQ-5D-3-L instead of the more sensitive 5 L version, as the 5 L was not available when the study was designed. Many patients with mRS changes already had one or more EQ-5D dimensions at an extreme level by V1–V3, consequently constraining the measurement of further change (See eFigure 10). Second, reliance on the mRS as a single anchor may be a limitation. Although multiple anchors are generally recommended, a single, well-validated anchor can be sufficient [45]. The mRS was selected due to its established use in stroke MID studies and strong correlation with the EQ-5D, whereas the available EQ-VAS possesses weaker validity as an anchor [16, 18].

### Implications

The application of phase-specific MIDs should differ by recovery stage—a principle established in guidelines for assessing meaningful change in quality of life in cancer patients [46]. Although derived from oncology, this principle is equally vital in stroke populations to distinguish between early and late disease stages. Specifically, acute‑phase values are most relevant for assessing short‑term treatment effects during admission, whereas subacute and chronic values are better suited for monitoring medium‑ to long‑term prognosis. To address these different clinical objectives, we report MIDs at both group and individual levels. For group-level evaluation, the MID can be determined for each TOAST subtype using its respective mean baseline score. Alternatively, for responder analysis at the individual level, a personalized MID may be calculated based on each patient’s specific baseline value [15].

Group‑level estimates are most informative for trial design and evidence interpretation. For example, the AC‑derived MID of 0.15 in the subacute phase can be used to calculate optimal information sizes and interpret whether the mean score change within a treatment group is clinically meaningful. Similarly, CD‑based estimates provide an alternative for between‑group comparisons. For clinicians interpreting evidence from clinical trials, observational studies, or meta-analyses, relative effects should be converted into absolute effects (ie, risk difference) to compared against the group-level MID values [47, 48]. We recommend the CD or group-pooled estimates (See eTable 2) as specific targets to determine whether a treatment effect is clinically important. These absolute thresholds are also essential for Core GRADE assessments. For instance, the certainty of evidence may be downgraded by one or two levels for imprecision, depending on whether the 95% CI crosses a single MID threshold or is wide enough to encompass both clinically meaningful improvement and deterioration [49]. Furthermore, as the MID is an absolute threshold, absolute rather than relative differences determine whether inconsistency is problematic [50].

Conversely, to determine if an individual patient’s utility change is clinically meaningful, the individual-level MID estimated via ROC analysis should be prioritized. We recommend using 0.10 (95% CI: 0.09 to 0.17) for the acute phase and 0.01 (95% CI: 0.01 to 0.10) for the subacute phase. Regarding the chronic phase, while the ROC analysis yielded an MID of 0.02, its reliability was considered borderline due to an AUC of 0.69 and a wide confidence interval approaching the null value (95% CI: 0.00 to 0.10). Consequently, this specific value was omitted from our primary reporting and should be applied with caution. Individual‑level estimates may help classify responders, although ROC‑derived thresholds in this study fell below corresponding MDC values and therefore carry some risk of misclassification (See eTable 4). Depending on clinical priorities, stricter cut‑points may increase specificity but reduce sensitivity.

Regarding variability across methods, anchor-based approaches (ie, AC-, CD-, ROC, and regression) are considered the most reliable because they directly link score changes to external clinical criteria. Distribution-based methods, by contrast, rely on statistical distribution and do not address the question of clinical importance. Consequently, these distribution-based approaches are reserved for cases where anchor-based calculations are unavailable or to serve as supportive benchmarks to validate and reinforce anchor-based estimates. In our study, the distribution-based pooled estimates closely aligned with the anchor-based pooled results, further validating the robustness of our results. Additionally, the instrument-defined approach acts as an anchor-based variant by using internal scale transitions within the value set as reference points. Given that MIDs should be disease-specific, such transitions should be interpreted with caution.

## Conclusion

This study establishes phase-specific MIDs for the EQ-5D after IS. At the group level, we recommend anchor-based pooled MIDs as they directly align score changes with clinical recovery. Based on this method, MIDs for improvement were 0.190 (0.17 to 0.21) for the acute phase, 0.14 (0.13 to 0.16) for the subacute phase, and 0.11 (0.10, 0.13) for the chronic phase, respectively. In clinical trials, change in EQ‑5D scores should be interpreted against these MIDs, with the most robust evidence obtained when changes are both statistically significant and clinically meaningful. Overall, the use of multiple methods to evaluate MID robustness in this analysis provides a reference for future research.

## Electronic supplementary material

Below is the link to the electronic supplementary material.

Supplementary Material 1

Supplementary Material 2

## Data Availability

The datasets used and/or analysed during the current study are available from the corresponding author on reasonable request.

## Abbreviations

AC: Average change
AIS: Acute ischemic stroke
AUC: Area under the curve
CASTOR: Chinese Acute Ischemic Stroke Treatment Outcome Registry
CD: Change difference
CE: Cardioembolic infarction
EQ-VAS: EQ-5D visual analogue scale
HRQOL: Health-related quality of life
LAA: Large-artery atherosclerosis
MDC: Minimal detectable change
MID: Minimally important difference
mRS: Modified Rankin Scale
NIHSS: National Institutes of Health Stroke Scale
ROC: Receiver operating characteristic
SA: Small-artery occlusion
SEM: Standard error of measurement
SOE: Stroke of other determined etiology
SUE: Stroke of undetermined etiology
TOAST: Trial of Org 10,172 in Acute Stroke Treatment.
