# Supplementary material for: Interpreting patient-reported outcomes after ischemic stroke: defining minimal important difference in EQ-5D across recovery phases
Source: Health Qual Life Outcomes. 2026 Mar 21;24:57. doi: 10.1186/s12955-026-02524-w (PMC13130762; doi:10.1186/s12955-026-02524-w)
Supplement: Supplementary file 1 — Supplementary Material 1 [file 12955_2026_2524_MOESM1_ESM.docx]

**SUPPLEMENTARY MATERIAL**

**1. Supplementary Material A — Distribution-based MID Estimation Methods**

| **Abbr.** | **Full Name** | **Formula/ Definition** | **Notes** | **Ref.** |
| --- | --- | --- | --- | --- |
| **ES** | Effect size | $ES=\frac{X_{1}-X_{0}}{\sqrt{{\sum\left( X_{0}-\bar{X_{0}} \right)^{2}}/\left( n-1 \right)}}$ | Mean change between baseline and follow-up, divided by the baseline SD, representing the “signal-to-noise ratio.” An ES of 0.5 was used as the threshold for a “small” effect. | Cohen, 1988[1] and Kazis et al, 1989[2] |
| **SD** | Standard deviation | $SD=\sqrt{\frac{1}{n-1}\sum_{i=1}^{n} \left( X_{0}-\bar{X_{0}} \right)^{2}}$ | Baseline SD. | Normanet al., 2003[3] |
| **SEM** | Standard error of measurement | ${SEM=SD}_{Baseline}\sqrt{1-r}$ | Calculated at baseline and follow-up, reflecting the extent of variation attributable to instrument unreliability. *r* represents the test-retest reliability coefficient. | Wyrich et al., 1999[4] |
| **MDC** | Minimal detectable change | ${MDC}_{ind}=SEM*z_{score}*\sqrt{2}$  ${MDC}_{group}={MDC}_{ind}\div\sqrt{n}$  $Ratio=MID\div MDC$ | A z-score of 1.64 corresponds to a 90% confidence level, and a z-score of 1.96 corresponds to a 95% confidence level. | Schmitt et al., 2007[5] and de Boer et al., 2005 [6] |

x_0_ = pre-test score; x_1_ = post-test score; n = sample size.

**REFERENCES**

1. J C: *Statistical power analysis for the behavioural sciences(2nd Edition).* NJ: Lawrence Erlbaum Associates; 1988.

2. Kazis LE, Anderson JJ, Meenan RF: Effect sizes for interpreting changes in health status. *Med Care* 1989, 27:S178-189.

3. Norman GR, Sloan JA, Wyrwich KW: Interpretation of changes in health-related quality of life: the remarkable universality of half a standard deviation. *Med Care* 2003, 41:582-592.

4. Wyrwich KW, Tierney WM, Wolinsky FD: Further evidence supporting an SEM-based criterion for identifying meaningful intra-individual changes in health-related quality of life. *J Clin Epidemiol* 1999, 52:861-873.

5. Schmitt JS, Di Fabio RP: Reliable change and minimum important difference (MID) proportions facilitated group responsiveness comparisons using individual threshold criteria. *J Clin Epidemiol* 2004, 57:1008-1018.

6. de Boer MR, de Vet HC, Terwee CB, Moll AC, Völker-Dieben HJ, van Rens GH: Changes to the subscales of two vision-related quality of life questionnaires are proposed. *J Clin Epidemiol* 2005, 58:1260-1268.

**2. Supplementary Material B — Supplementary Tables and Figures**

**eTable 1.** Distribution of EQ-5D-3L utility index and modified Rankin scale scores.

| **Distribution of** **EQ-5D Measurement Results** | | | | | | | | | |
| --- | --- | --- | --- | --- | --- | --- | --- | --- | --- |
|  | **Scale** | ***N*** | | **Mean (SD)** | **Median** | | **Max** | | **Min** |
| V1 | EQ-5D-3L | 9998 | | 0.546 (0.327) | 0.597 | | 1.00 | | -0.149 |
|  | EQ-VAS |  |  | 64.9 (19.8) | 70 | | 100 | | 0 |
| V2 | EQ-5D-3L | 9980 | | 0.709 (0.306) | 0.783 | | 1.00 | | -0.149 |
|  | EQ-VAS |  |  | 78.1 (17.4) | 80 | | 100 | | 0 |
| V3 | EQ-5D-3L | 9264 | | 0.801 (0.259) | 0.875 | | 1.00 | | -0.149 |
|  | EQ-VAS |  |  | 83.1 (15.1) | 90 | | 100 | | 0 |
| V4 | EQ-5D-3L | 8576 | | 0.846 (0.237) | 1.00 | | 1.00 | | -0.149 |
|  | EQ-VAS |  |  | 85.8 (14.2) | 90 | | 100 | | 0 |
| **Distribution of mRS Scores Results** | | | | | | | | | |
|  | **V1, *N* (%)**  Phase | | **V2, *N* (%)** | | | **V3, *N* (%)** | | **V4, *N* (%)** | |
| 0  Rating | 453 (4.53) | | 1501 (15.04) | | | 2108 (22.75) | | 2547 (29.70) | |
| 1 | 3166 (31.67) | | 3821 (38.29) | | | 3801 (41.03) | | 3497 (40.78) | |
| 2 | 2073 (20.73) | | 1682 (16.85) | | | 1507 (16.27) | | 1257 (14.66) | |
| 3 | 1333 (13.33) | | 1122 (11.24) | | | 939 (10.14) | | 648 (7.56) | |
| 4 | 1890 (18.90) | | 1256 (12.59) | | | 639 (6.90) | | 415 (4.84) | |
| 5 | 1083 (10.83) | | 597 (5.98) | | | 264 (2.85) | | 209 (2.44) | |
| 6 | 0 (0.00) | | 1 (0.01) | | | 6 (0.06) | | 3 (0.03) | |

Abbreviations: mRS, Modified Rankin Scale (range 0-6).

***Note.*** V1, hospital admission; V2, hospital discharge; V3, 90 ± 14 d post-admission; V4, 360 ± 28 d post-admission. mRS grades: 0, asymptomatic; 1, slight symptoms; 2, slight disability (independent); 3-5, moderate-to-severe disability (3, moderate; 4, moderately severe; 5, severe); 6, death.

**eTable 2.** MIDs for EQ-5D-3L from the anchor-, distribution-, and instrument-based methods.

|  | **EQ-5D-3L Utility Index** | | | | | | |
| --- | --- | --- | --- | --- | --- | --- | --- |
| **Direction of Change** | **Method** | **V2** | **95%CI**^a^ | **V3** | **95%CI** | **V4** | **95%CI** |
| **All** | Anchor-based | | | | | | |
|  | AC | 0.180 | 0.174, 0.187 | 0.104 | 0.096, 0.111 | 0.065 | 0.058, 0.073 |
|  | CD | 0.100 | 0.092, 0.107 | 0.068 | 0.061, 0.076 | 0.044 | 0.037, 0.052 |
|  | Regression | 0.217 | 0.212, 0.222 | 0.157 | 0.152, 0.162 | 0.129 | 0.124, 0.133 |
|  | Pooled_group_ | 0.166 | 0.147, 0.185 | 0.110 | 0.092, 0.127 | 0.079 | 0.062, 0.096 |
|  | ROC | NA | NA | NA | NA | NA | NA |
|  | Distribution-based | | | | | | |
|  | 0.2SD | 0.065 | NA | 0.061 | NA | 0.052 | NA |
|  | 0.5SD | 0.163 | NA | 0.153 | NA | 0.129 | NA |
|  | SEM | 0.180 | NA | 0.163 | NA | 0.139 | NA |
|  | 0.5ES | 0.249 | NA | 0.150 | NA | 0.087 | NA |
|  | Pooled | 0.164 | NA | 0.132 | NA | 0.102 | NA |
|  | Instrument-defined^b^ | | | | | | |
|  | idMID | 0.120 | NA | 0.108 | NA | 0.108 | NA |
| **Improve** | Anchor-based | | | | | | |
|  | AC | 0.216 | 0.209, 0.222 | 0.153 | 0.145, 0.160 | 0.119 | 0.111, 0.126 |
|  | CD | 0.135 | 0.128, 0.143 | 0.117 | 0.109, 0.125 | 0.098 | 0.090, 0.106 |
|  | Regression | 0.216 | 0.210, 0.221 | 0.153 | 0.147, 0.158 | 0.119 | 0.113, 0.125 |
|  | Pooled_group_ | 0.189 | 0.172, 0.206 | 0.141 | 0.125, 0.157 | 0.112 | 0.096, 0.128 |
|  | ROC | 0.104 | 0.093, 0.168 | 0.007 | 0.006, 0.096 | NA | NA |
|  | Distribution-based | | | | | | |
|  | 0.2SD | 0.066 | NA | 0.059 | NA | 0.050 | NA |
|  | 0.5SD | 0.165 | NA | 0.147 | NA | 0.126 | NA |
|  | SEM | 0.169 | NA | 0.146 | NA | 0.124 | NA |
|  | 0.5ES | 0.286 | NA | 0.139 | NA | 0.102 | NA |
|  | Pooled | 0.172 | NA | 0.123 | NA | 0.101 | NA |
|  | Instrument-defined | | | | | | |
|  | idMID | 0.091 | NA | 0.093 | NA | 0.093 | NA |
| **Deteriorate** | Anchor-based | | | | | | |
|  | AC | 0.086 | 0.063, 0.109 | 0.084 | 0.071, 0.098 | 0.087 | 0.073, 0.100 |
|  | CD | 0.075 | 0.058, 0.092 | 0.053 | 0.041, 0.065 | 0.065 | 0.054, 0.076 |
|  | Regression | 0.086 | 0.071, 0.101 | 0.084 | 0.073, 0.095 | 0.087 | 0.078, 0.096 |
|  | Pooled_group_ | 0.082 | 0.064, 0.101 | 0.074 | 0.063, 0.085 | 0.080 | 0.068, 0.091 |
|  | ROC | NA | NA | NA | NA | NA | NA |
|  | Distribution-based | | | | | | |
|  | 0.2SD | 0.066 | NA | 0.069 | NA | 0.055 | NA |
|  | 0.5SD | 0.164 | NA | 0.172 | NA | 0.137 | NA |
|  | SEM | 0.168 | NA | 0.164 | NA | 0.132 | 0.168 |
|  | 0.5ES | 0.071 | NA | 0.215 | NA | 0.079 | NA |
|  | Pooled | 0.117 | NA | 0.155 | NA | 0.101 | NA |
|  | Instrument-defined | | | | | | |
|  | idMID | 0.148 | NA | 0.123 | NA | 0.123 | NA |

Abbreviations: CI, confidence interval; AC, average change; CD, change difference; ROC, receiver operating characteristic; ES, effect size; NA, not applicable; SD, standard deviation; SEM, standard error of measurement; idMID, instrument-defined minimally important difference.

^a^ Interval estimation was derived by using bootstrapping with 5000 replicates. Pooled CI estimates combine individual SE^2^ and observed MlD variance under-distribution, Values for direction of change deteriorate have been multiplied by -1.

^b^ The method of Instrument-defined was used to calculate the baseline, which V2 is the same as V3.

***Note.*** V2, V3, and V4 represent the acute (V1–V2), subacute (V2–V3), and chronic (V3–V4) phases, respectively.

**eTable 3.** Sample sizes used for calculating anchor- and distribution-based methods.

| **Method** | **Direction of Change** | **Sample size, *N* (%)** | | |
| --- | --- | --- | --- | --- |
|  |  | **V2 vs. V1** | **V3 vs. V2** | **V4 vs. V3** |
| **Anchor-based** | Minor change | 3650 (44.4) | 2909 (35.6) | 2374 (30.3) |
|  | Improve | 3220 (39.2) | 2312 (28.3) | 1756 (22.4) |
|  | Deteriorate | 430 (5.2) | 597 (7.3) | 618 (7.9) |
|  | No change | 4570 (55.6) | 5254 (64.4) | 5453 (69.7) |
|  | Sum | 8220 (100) | 8163 (100) | 7827 (100) |
| **Distribution-based** | Major change | 1724 (17.3) | 999 (10.9) | 569 (6.8) |
|  | Improve | 1457 (14.7) | 826 (9) | 375 (4.5) |
|  | Deteriorate | 267 (2.7) | 173 (1.9) | 194 (2.3) |
|  | Minor change | 3650 (36.7) | 2909 (31.8) | 2374 (28.3) |
|  | Improve | 3220 (32.4) | 2312 (25.2) | 1756 (20.9) |
|  | Deteriorate | 430 (4.3) | 597 (6.5) | 618 (7.4) |
|  | No change | 4570 (46) | 5254 (57.3) | 5453 (64.9) |
|  | Sum | 9944 (100) | 9162 (100) | 8396 (100) |

**eTable 4.** Performance of different MDC and MID by ROC at the individual level.

| **Direction** | **Phase** | | **Measure** | **Cut-point** | **Sensitivity,** % | **Specificity,** % | **PPV,** % | **NPV,** % |
| --- | --- | --- | --- | --- | --- | --- | --- | --- |
| Improve | | Acute phase | MDC 95 | 0.468 | 23 | 98 | 92 | 45 |
|  |  |  | MDC 90 | 0.392 | 29 | 97 | 90 | 43 |
|  |  |  | MID individual | 0.104 | 78 | 64 | 69 | 26 |
|  |  | Subacute phase | MDC 95 | 0.405 | 18 | 98 | 87 | 33 |
|  |  |  | MDC 90 | 0.339 | 25 | 98 | 86 | 31 |
|  |  |  | MID individual | 0.007 | 73 | 69 | 59 | 19 |
|  |  | Chronic phase | MDC 95 | 0.344 | 13 | 99 | 84 | 25 |
|  |  |  | MDC 90 | 0.288 | 24 | 97 | 78 | 23 |
|  |  |  | MID individual | NA |  |  |  |  |
| Deteriorate | | Acute phase | MDC 95 | 0.466 | 17 | 100 | 94 | 11 |
|  |  |  | MDC 90 | 0.390 | 27 | 100 | 94 | 10 |
|  |  |  | MID individual | NA |  |  |  |  |
|  |  | Subacute phase | MDC 95 | 0.455 | 11 | 100 | 79 | 12 |
|  |  |  | MDC 90 | 0.380 | 15 | 99 | 74 | 11 |
|  |  |  | MID individual | NA |  |  |  |  |
|  |  | Chronic phase | MDC 95 | 0.366 | 17 | 99 | 82 | 11 |
|  |  |  | MDC 90 | 0.306 | 23 | 99 | 78 | 10 |
|  |  |  | MID individual | NA |  |  |  |  |

Abbreviations: MDC, meaningful detectable change; MID, minimal important difference; ROC, receiver operator characteristic curve; AUC, area under the curve; PPV, Positive predictive value; NPV, Negative predictive value.

***Note.*** The ROC with an AUC below 0.7 was not reliable and was excluded from the analysis.

**eTable 5.** Credibility Assessment Results.

| **CREDIBILITY ASSESSMENT - CORE CRITERIA** |
| --- |
| **Is the patient or necessary proxy responding directly to both the PROM and the anchor?**  *If a* *clinician* *or* *anyone* *else* *is* *responding* *to* *the* *anchor* *directly* *and* *the* *patients* *are* *capable* *of* *providing* *this* *information,* *the* *answer* *should* *be* *"no."* *Any* *other* *necessary* *proxy* *(e.g.* *caregiver,* *parent,* *wife,* *relative)* *responding* *to* *the* *anchor,* *the* *answer* *is "yes".*  [ X ] Yes [ ] No [ ] Impossible to tell  Rationale: The data collection methods for PROMs and anchor measures in this study were as follows: admission and discharge follow-ups were conducted via face-to-face patient interviews, while all subsequent follow-ups were performed through telephone interviews with patients. |
| **Is the anchor easily understandable and relevant for patients or necessary proxy?**  *With* *"easily* *understandable* *and* *relevant"* *we* *mean* *that,* *when* *presented* *with* *the* *anchor* *(either* *actually* *presented* *or* *hypothetically)* *as* *an outcome,* *and* *without* *too* *much* *education,* *the* *patients* *are* *able* *to* *understand* *the* *data* *provided* *for* *the* *outcome* *(anchor)* *and* *use* *it* *easily* *for* *decision-making.* *For* *example,* *when* *addressing* *a* *multi-item* *PROM* *addressing* *the* *potential* *therapeutic* *effects* *of* *an* *intervention* *for* *iron-deficiency* *anemia,* *an* *anchor* *of* *patient’s* *global* *rating* *of* *improvement* *in* *fatigue* *may* *be* *easier* *to* *understand* *and* *more* *relevant* *for* *decision-* *making* *than* *serum* *iron* *levels.* *If* *you* *were* *a* *patient,* *how* *would* *you* *answer* *this* *question?*  [ X ] Definitely yes [ ] To a great extent [ ] Not so much [ ] Definitely no [ ] Impossible to tell  Rationale: The anchor mRS ratings selected for this study demonstrate high clinical relevance, simplicity of use, and robust reliability and validity. |
| **Has the anchor shown good correlation with the PROM?**  *This* *assessment* *is* *made* *using* *the* *correlation* *coefficients* *reported* *by* *the* *authors.* *If* *the* *anchor* *is* *a* *transition* *question* *then* *this* *is* *correlation* *between* *the* *transition* *item* *and* *the* *PROM* *change* *score.* *For* *any* *other* *anchor,* *this* *is* *the* *correlation* *between* *the* *change* *in* *the* *anchor* *and* *the* *change* *in* *the* *PROM.* *If* *the* *study* *is* *cross-sectional,* *this* *is* *the* *correlation* *between* *the* *anchor* *and* *the* *PROM* *score.* *Only* *consider* *the* *absolute* *value* *of* *the* *correlation* *coefficient.*  [ ] Definitely yes [ X ] To a great extent [ ] Not so much [ ] Definitely no [ ] Impossible to tell  ≥0.7 ≥0.5 to <0.7 ≥0.3 to <0.5 <0.3  Rationale: The anchors selected in this study demonstrated strong correlations with the outcome measures. Specifically, the absolute values of correlation coefficients were: ≥0.606 for all changes, ≥0.561 for improve changes, and ≥0.355 for deteriorate changes. |
| **Is the MID precise?**  *Precision* *around* *the* *MID* *estimate* *is* *quantified* *by* *the* *width* *of* *the* *95%* *CI* *and* *expressed* *as* *a* *percentage.* *For* *example,* *if* *the* *MID* *estimate* *is* *23.5* *and* *the* *95%* *CI* *ranges* *from* *23.1* *to* *23.8,* *then* *precision* *may* *be* *calculated* *as:* *23.8* *一* *23.1* */* *23.5* *** *100* *=* *3%.* *According* *to* *our* *guide* *provided* *for* *our* *responses* *to* *this* *credibility* *question,* *a* *result* *of* *3%* *would* *warrant* *a* *rating* *of* *definitely* *yes.* *In* *many* *cases,* *the* *authors* *may* *not* *report* *any* *measure* *of* *variability* *(e.g.* *SD,* *SE,* *95%* *CI).* *In* *these* *situations,* *we* *ask* *that* *you* *consider* *the* *sample* *size* *used* *to* *estimate* *the* *MID.* *We* *provide* *ranges* *for* *both* *situations* *(i.e.* *percentage* *of* *the* *confidence* *interval* *width* *in* *relation* *to* *the* *MID,* *and* *sample* *sizes)* *to* *help* *inform* *your* *judgment.* *If* *the* *judgments* *according* *to* *the* *two* *criteria* *differ,* *we* *suggest* *using* *the* *higher* *(more* *permissive)* *of* *the* *two* *ratings.*  [ X ] Definitely yes [ ] To a great extent [ ] Not so much [ ] Definitely no [ ] Impossible to tell  ≤10% **or** ≥200 patients 11-25% **or** 150-199 patients 26-49% **or** 100-149 patients ≥50% **or** <100 patients  Rationale: The study calculated both the percentage of the confidence interval width relative to the MID and the sample size, ultimately selecting the more lenient criterion. The results showed that the study included a sample size of 9,978 participants, which far exceeds the threshold of 200 patients. |
| **Does the threshold or difference between groups on the anchor used to estimate the MID reflect a small but important difference?**  *Establishing the degree of change on a PROM that constitutes the MID requires some knowledge about the degree of change on the anchor that is small but important to patients. In addition to inspecting the threshold on the anchor, it is necessary to judge whether the method of analysis indeed calculates a small but important difference. Below, we present examples and provide associated guidance.*  *For transition rating anchors, consider the wording and number of responses. For instance, the mean change in PROM score in patients with a transition rating anchor scale designation of (a little better’ on a seven-point scale including the categories (much worse, somewhat worse, a little worse, no change, a little better, somewhat better, much better, ’ as reflecting an MlD would warrant a definitely yes, whereas a choice of "much better" would warrant a definitely no.*  *In* *some* *cases,* *authors* *may* *use* *a* *threshold* *for* *their* *analysis* *and* *include* *only* *patients* *who* *achieved* *this* *threshold;* *other* *times,* *they* *may* *include* *patients* *who* *achieved* *this* *threshold* *or* *greater.* *For* *instance,* *the* *investigators* *may* *define* *the* *MID* *as* *the* *mean* *change* *in* *the* *PROM* *score* *in* *patients* *who* *achieved* *a* *≥5%* *change* *in* *weight* *loss. This approach includes even those patients who had a 10%, 30% or 50% reduction in weight loss and thus would warrant a definitely no.*  [ X ] Definitely yes [ ] To a great extent [ ] Not so much [ ] Definitely no [ ] Impossible to tell  Rationale: In this large-scale prospective study, the threshold adopted a one-grade change in mRS ratings, which has demonstrated sensitivity in detecting clinically meaningful changes, as established through both the prospective study design and Delphi method validation. |

**eTable 6.** Kruskal-Wallis test results.

| **TOAST** | **V1 (N=4475)** | | **V2 (N=4461)** | | **V3 (N=4143)** | | **V4 (N=3746)** | |
| --- | --- | --- | --- | --- | --- | --- | --- | --- |
|  | Utility | No. (%) | Utility | No. (%) | Utility | No. (%) | Utility | No. (%) |
| SA | 0.678 | 1131 (25.3) | 0.797 | 1129 (25.3) | 0.864 | 1129 (25.3) | 0.905 | 1129 (25.3) |
| SOE | 0.610 | 159 (2.3) | 0.782 | 105 (2.4) | 0.846 | 105 (2.4) | 0.888 | 105 (2.4) |
| SUE | 0.591 | 2890 (3.6) | 0.763 | 158 (3.5) | 0.849 | 158 (3.5) | 0.883 | 158 (3.5) |
| LAA | 0.585 | 190 (64.6) | 0.693 | 2880 (64.6) | 0.787 | 2880 (64.6) | 0.845 | 2880 (64.6) |
| CE | 0.218 | 190 (4.2) | 0.505 | 189 (4.2) | 0.656 | 189 (4.2) | 0.734 | 189 (4.2) |
| **statistic** | 124.20 | | 124.20 | | 104.25 | | 104.25 | |
| ***P*** | <.001 | | <.001 | | <.001 | | <.001 | |

Abbreviations: SA, small-artery occlusion; SOE, stroke of other explicit etiology; SUE, stroke of undetermined etiology; LAA, large-artery atherosclerosis; CE, cardioembolic infarction.

**eTable 7.** Post-hoc Dunn-Bonferroni analysis.

| Visit | group1 | group2 | statistic | Adjusted *P* | Adjusted *P* significance |
| --- | --- | --- | --- | --- | --- |
| 1 | LAA | CE | -6.814 | 0.000 | **** |
| 1 | LAA | SA | 9.265 | 0.000 | **** |
| 1 | LAA | SOE | 2.652 | 0.000 | **** |
| 1 | LAA | SUE | 3.430 | 0.000 | **** |
| 1 | CE | SA | 10.650 | 0.000 | **** |
| 1 | CE | SOE | 6.369 | 0.058 | ns |
| 1 | CE | SUE | 7.346 | 0.437 | ns |
| 1 | SA | SOE | -0.606 | 0.761 | ns |
| 1 | SA | SUE | -0.531 | 0.761 | ns |
| 1 | SOE | SUE | 0.133 | 0.773 | ns |
| 2 | LAA | CE | -6.814 | 0.000 | **** |
| 2 | LAA | SA | 9.265 | 0.000 | **** |
| 2 | LAA | SOE | 2.652 | 0.080 | ns |
| 2 | LAA | SUE | 3.430 | 0.006 | ** |
| 2 | CE | SA | 10.650 | 0.000 | **** |
| 2 | CE | SOE | 6.369 | 0.000 | **** |
| 2 | CE | SUE | 7.346 | 0.000 | **** |
| 2 | SA | SOE | -0.606 | 1.000 | ns |
| 2 | SA | SUE | -0.531 | 1.000 | ns |
| 2 | SOE | SUE | 0.133 | 1.000 | ns |
| 3 | LAA | CE | -4.756 | 0.000 | **** |
| 3 | LAA | SA | 7.938 | 0.000 | **** |
| 3 | LAA | SOE | 2.606 | 0.092 | ns |
| 3 | LAA | SUE | 2.563 | 0.104 | ns |
| 3 | CE | SA | 7.998 | 0.000 | **** |
| 3 | CE | SOE | 5.090 | 0.000 | **** |
| 3 | CE | SUE | 5.274 | 0.000 | **** |
| 3 | SA | SOE | -0.176 | 1.000 | ns |
| 3 | SA | SUE | -0.784 | 1.000 | ns |
| 3 | SOE | SUE | -0.387 | 1.000 | ns |
| 4 | LAA | CE | -4.300 | 0.000 | *** |
| 4 | LAA | SA | 7.734 | 0.000 | **** |
| 4 | LAA | SOE | 2.169 | 0.301 | ns |
| 4 | LAA | SUE | 1.315 | 1.000 | ns |
| 4 | CE | SA | 7.377 | 0.000 | **** |
| 4 | CE | SOE | 4.471 | 0.000 | **** |
| 4 | CE | SUE | 4.027 | 0.001 | *** |
| 4 | SA | SOE | -0.497 | 1.000 | ns |
| 4 | SA | SUE | -1.833 | 0.669 | ns |
| 4 | SOE | SUE | -0.842 | 1.000 | ns |

Asterisks (*) indicate statistical significance: **P*<.05, ** *P*<.01, *** *P*<.001, **** *P*<.0001; non-significant results denoted as “ns”.

***Note.*** SA > LAA > CE across Vs 2-4; SUE and SOE comparisons not significant.


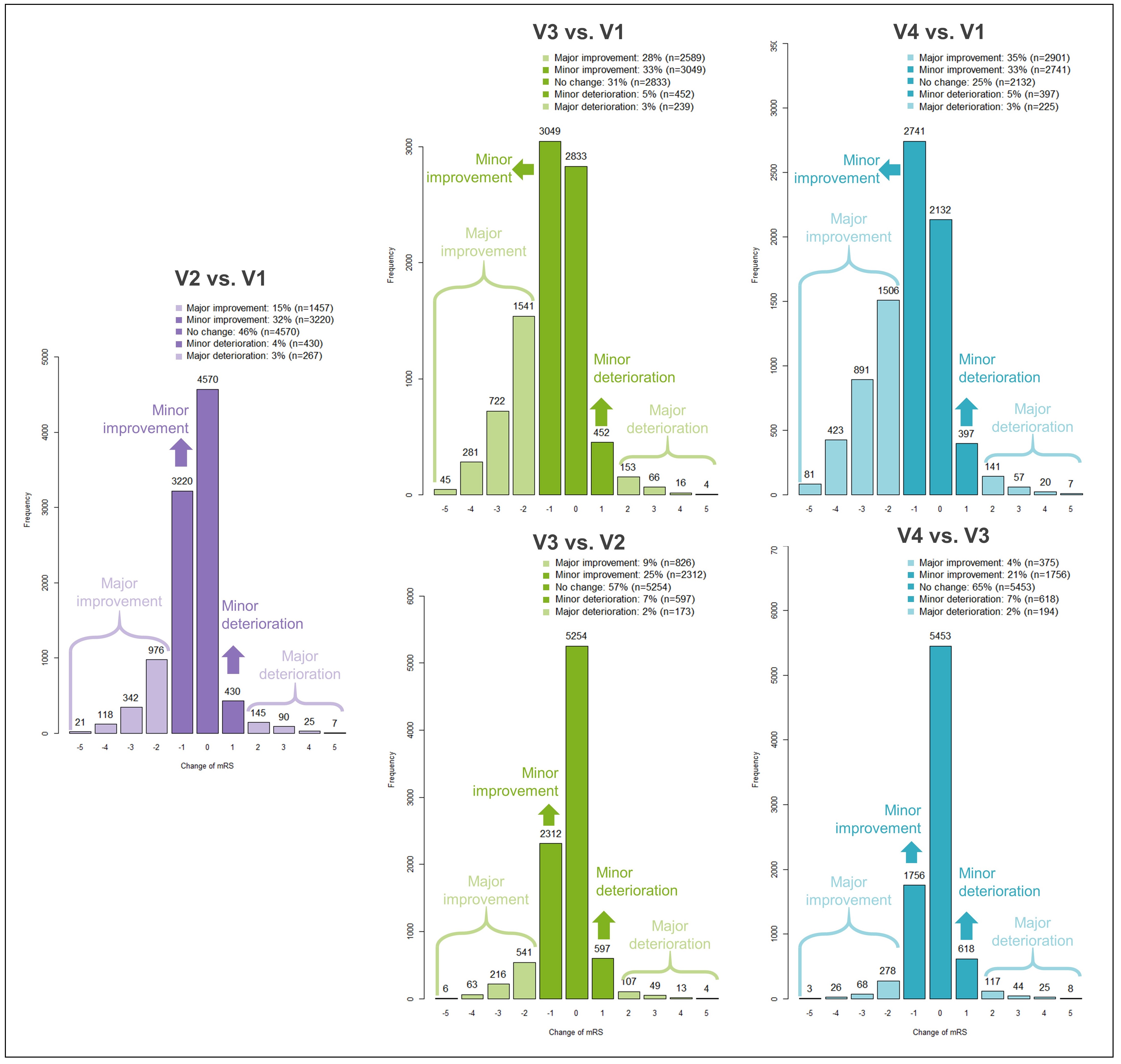


**eFigure 1.** Distribution of mRS score changes. (**Top**) Changes relative to baseline (V3/V4 vs. V1); (**Bottom**) Changes between adjacent study visits (V3 vs. V2 and V4 vs. V3).


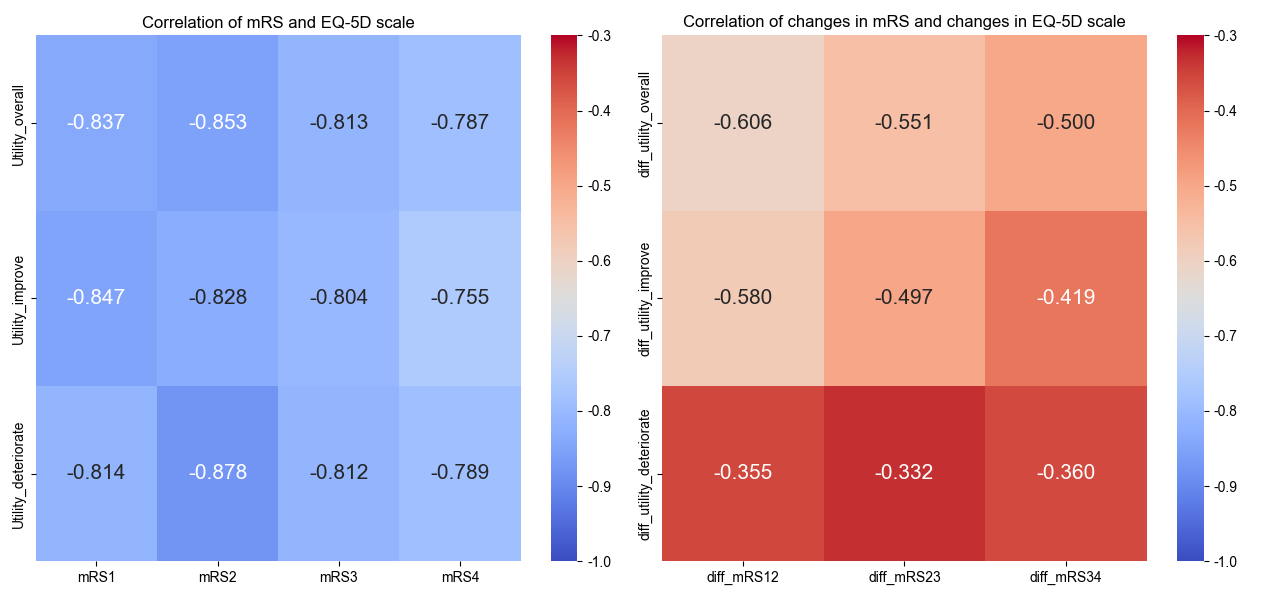


**eFigure 2.** Correlation between EQ-5D-3L scale with mRS (left) and change in EQ-5D scale with change in mRS (right).

***Note.*** The suffixes 2-4 of mRS represent Visits 2-4 respectively; 23 indicates the difference between V3 and V2, and 34 follow the same logic.


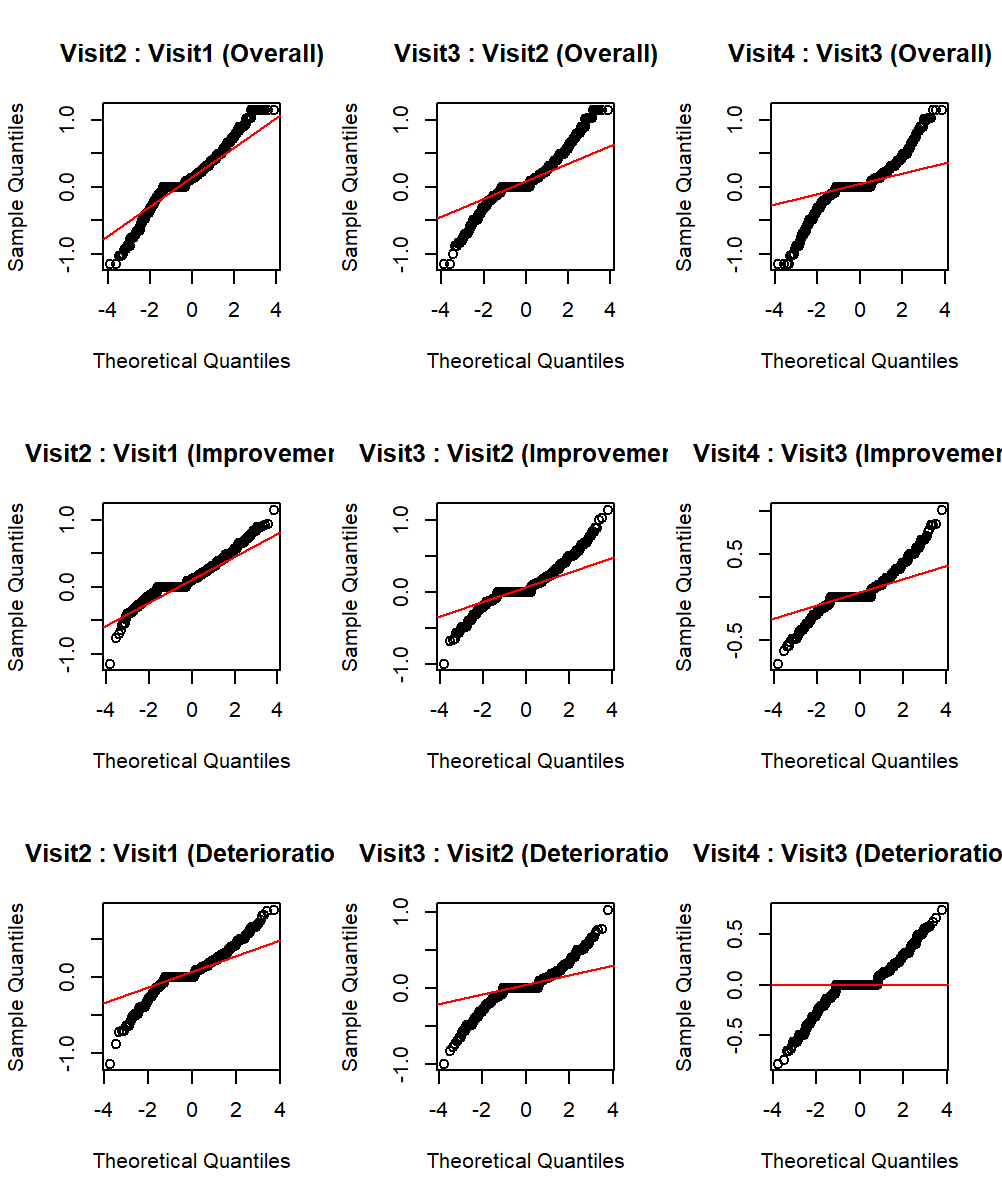


**eFigure 3.** Normal Q-Q plots assessing normality of Δutility.

**
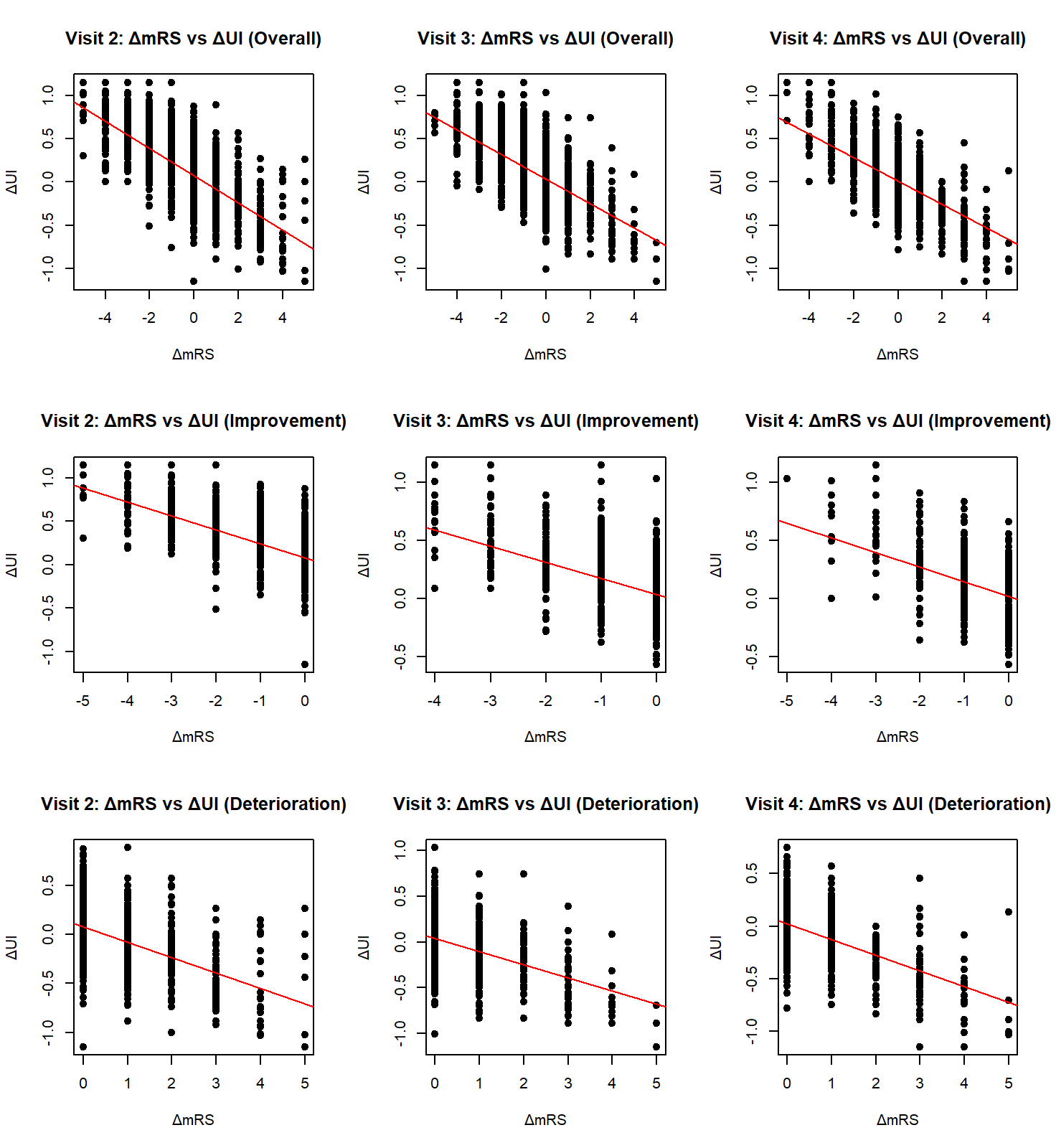
**

**eFigure 4.** Scatter plot of EQ-5D utility change vs. mRS score change.

| 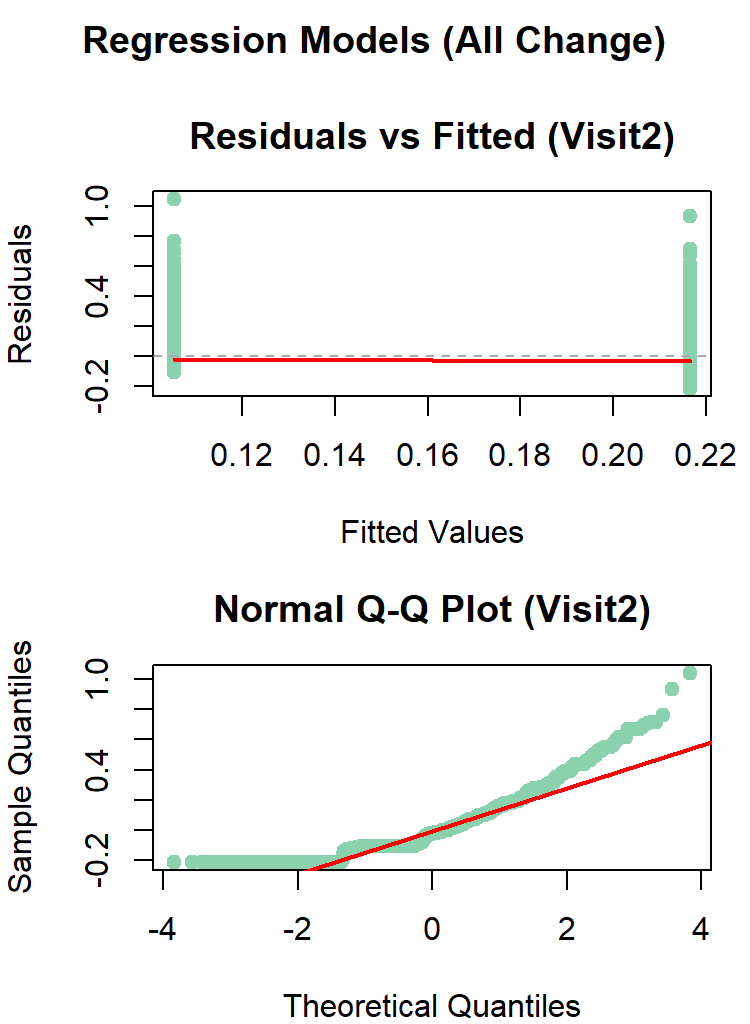 | 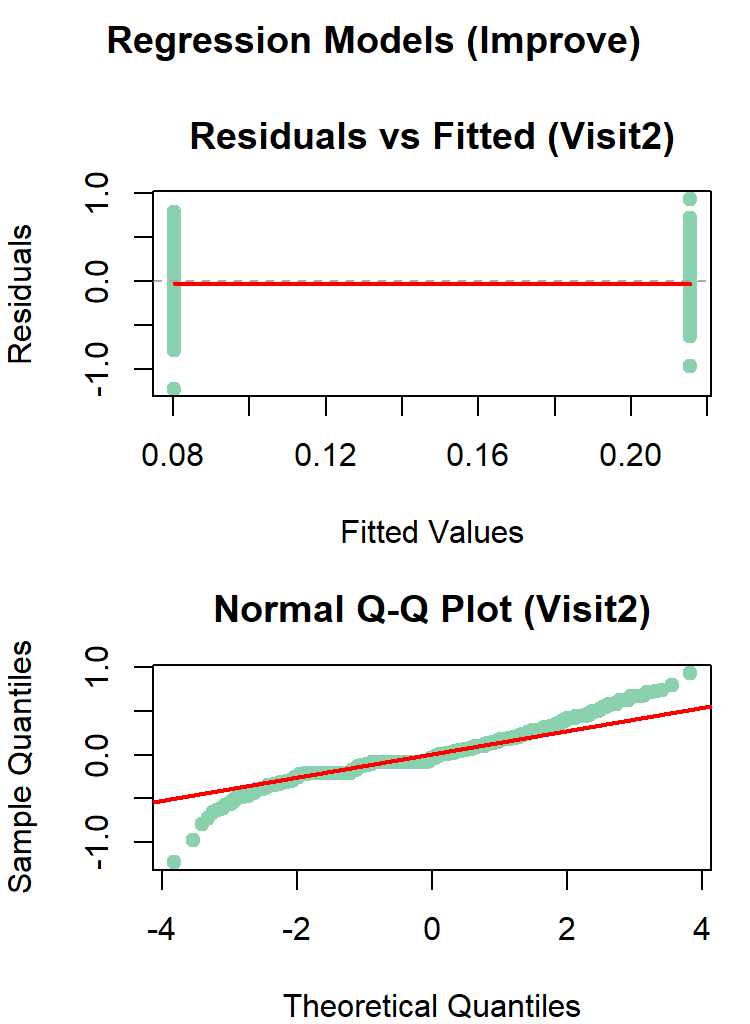 | 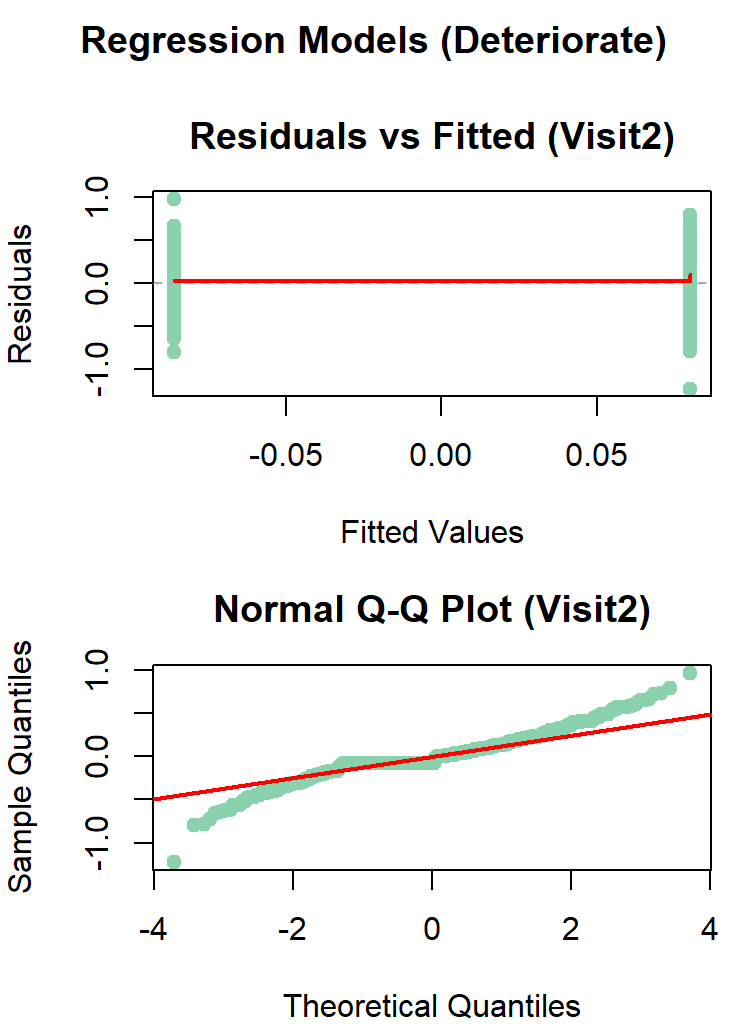 |
| --- | --- | --- |
| 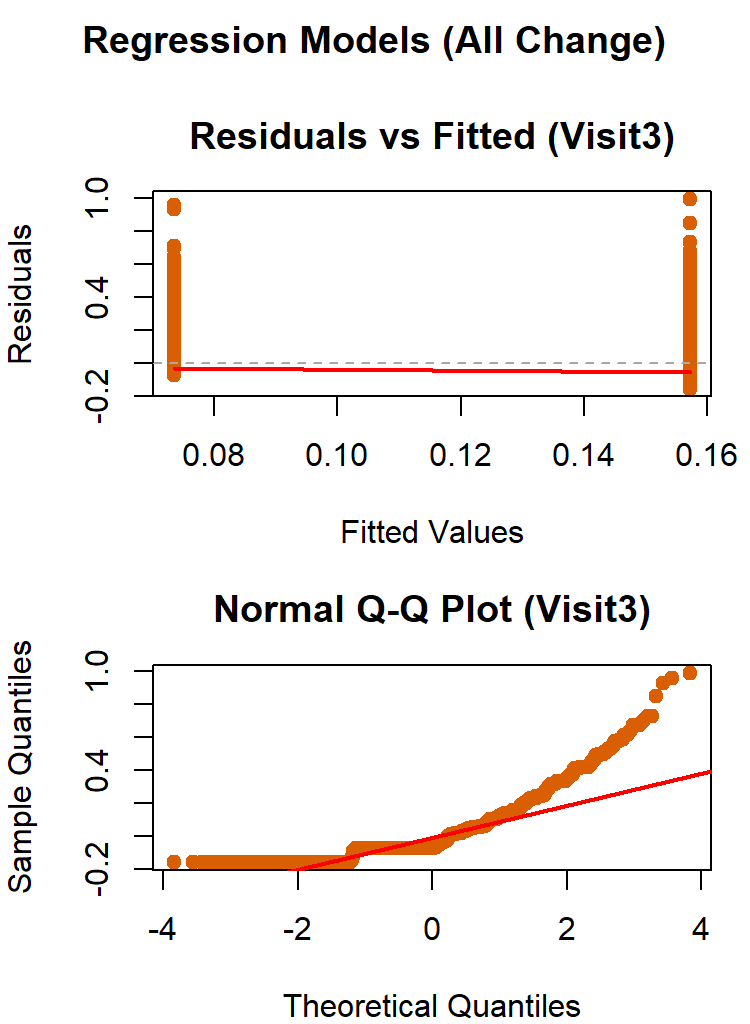 | 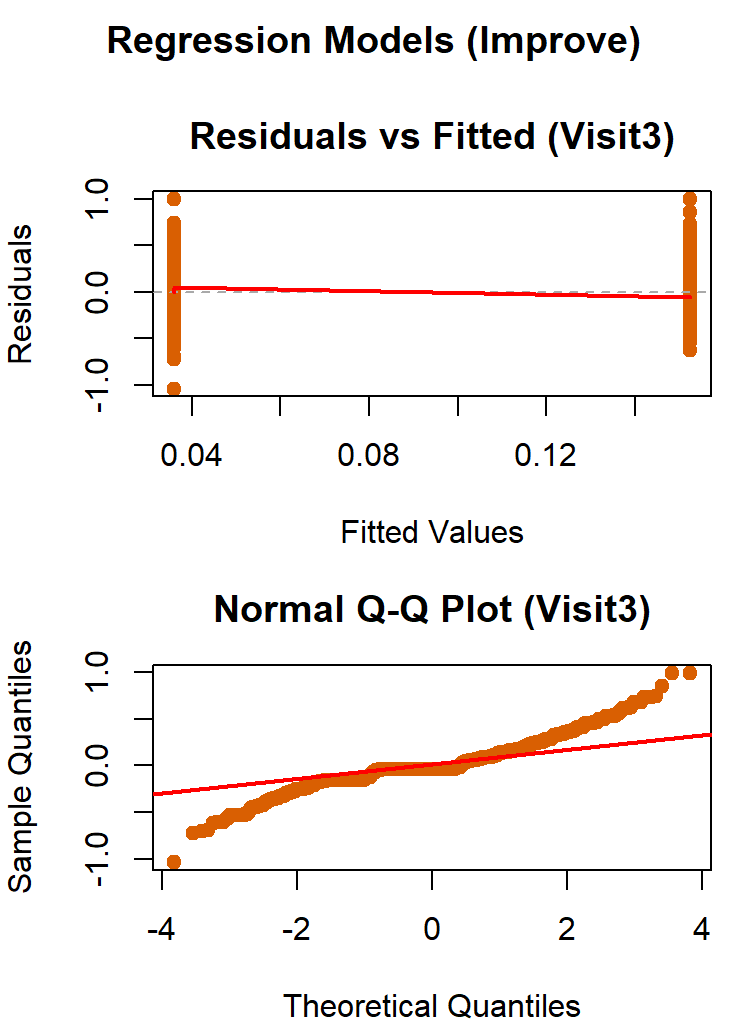 | 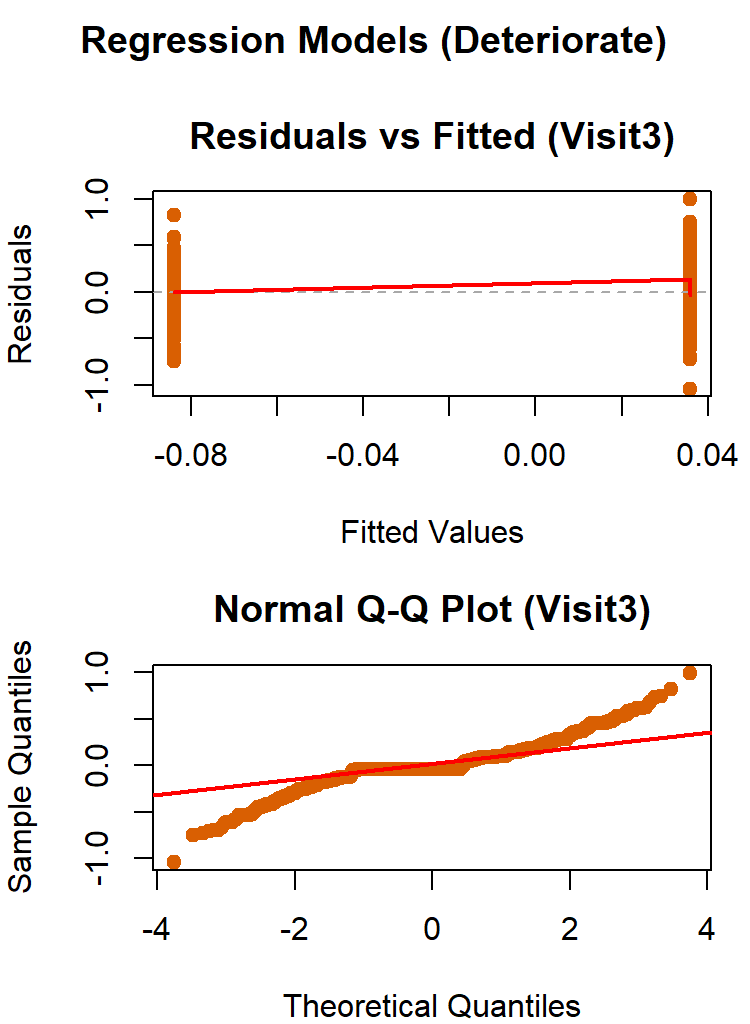 |
| 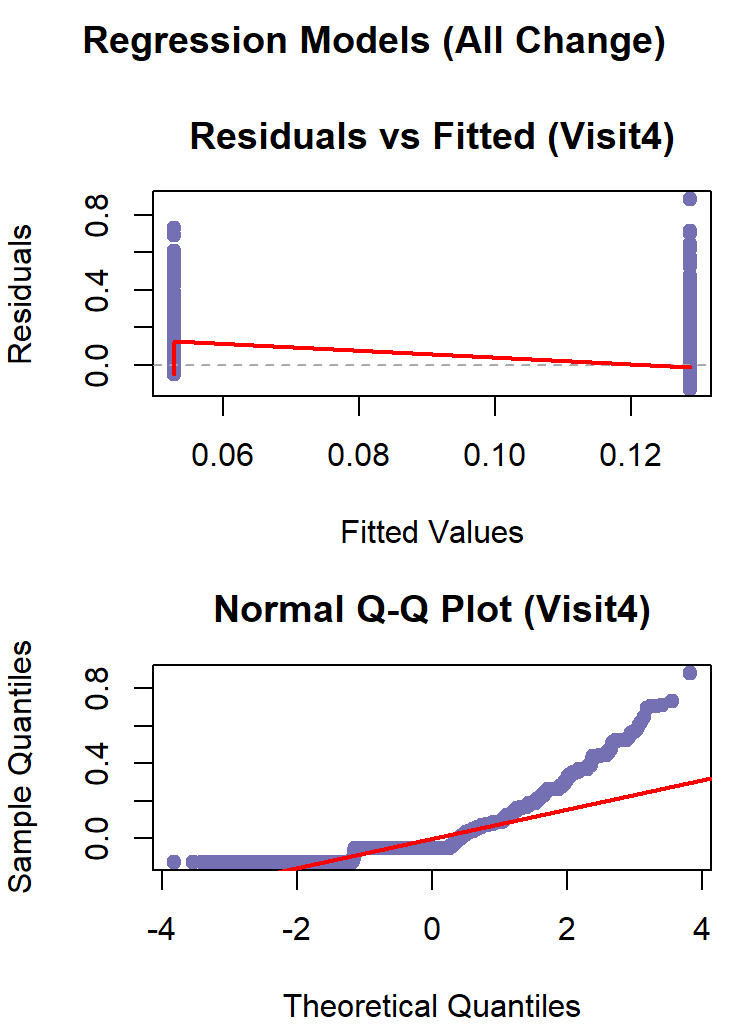 | 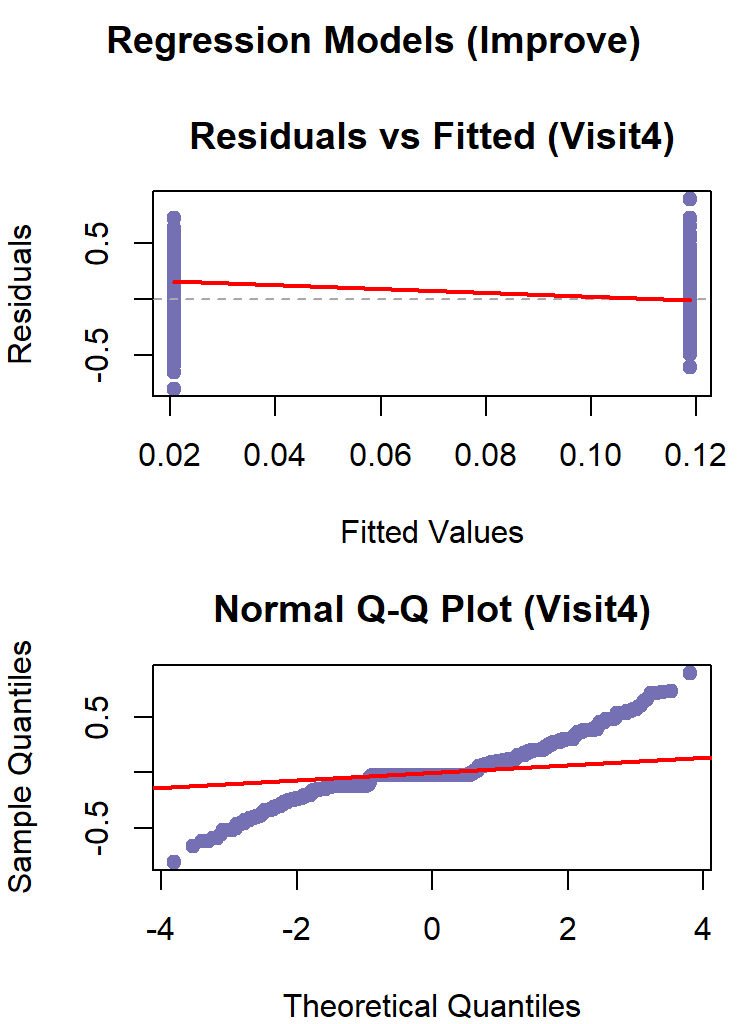 | 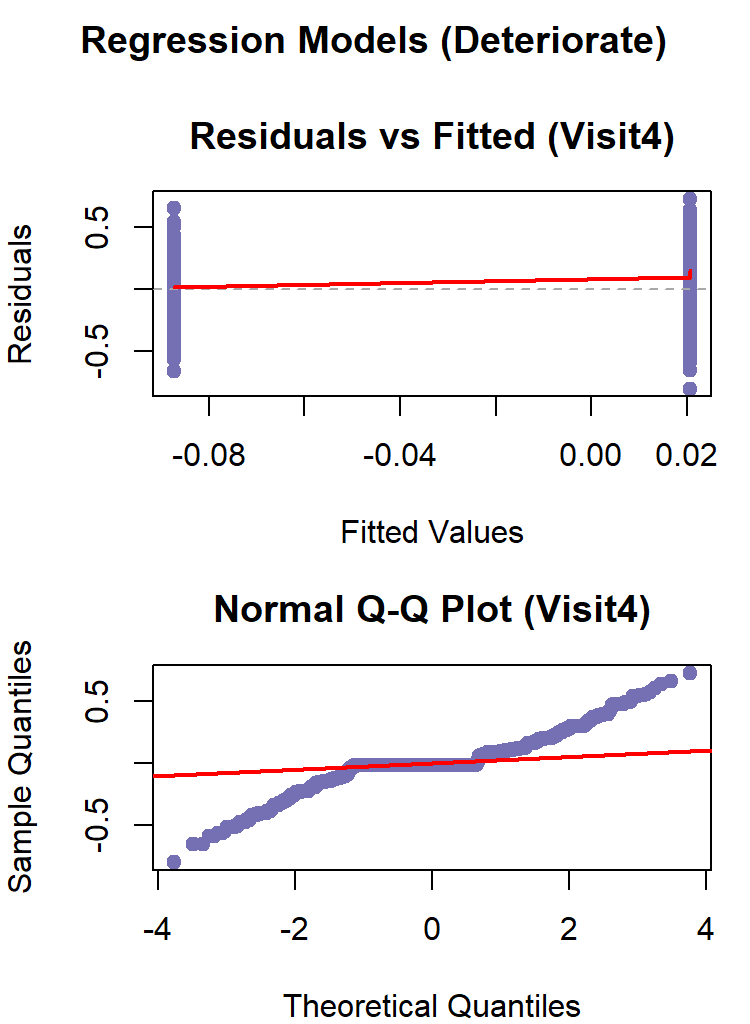 |

**eFigure 5.** Residuals versus fitted values and normal Q–Q plots for linear regression assumptions.


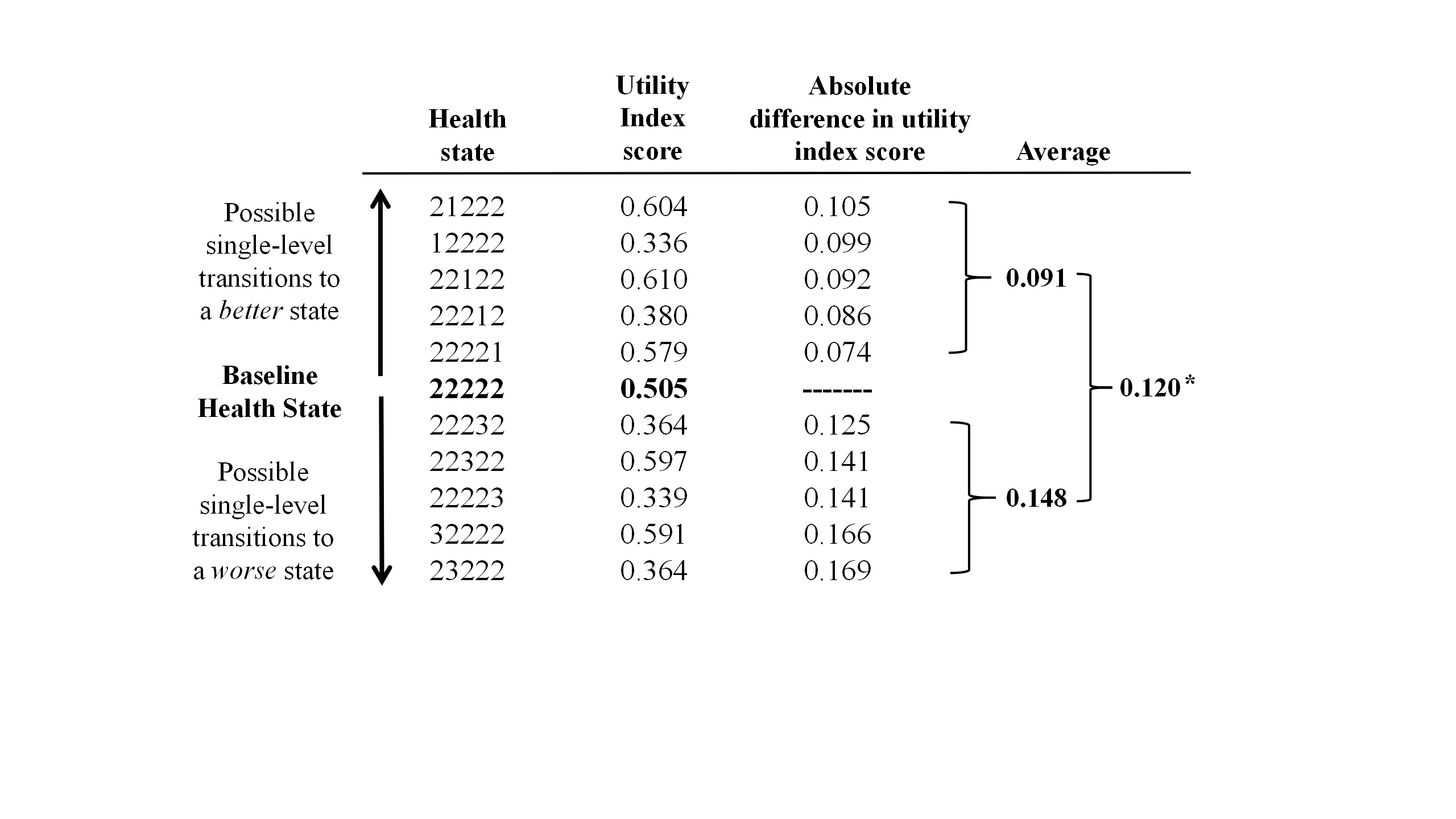

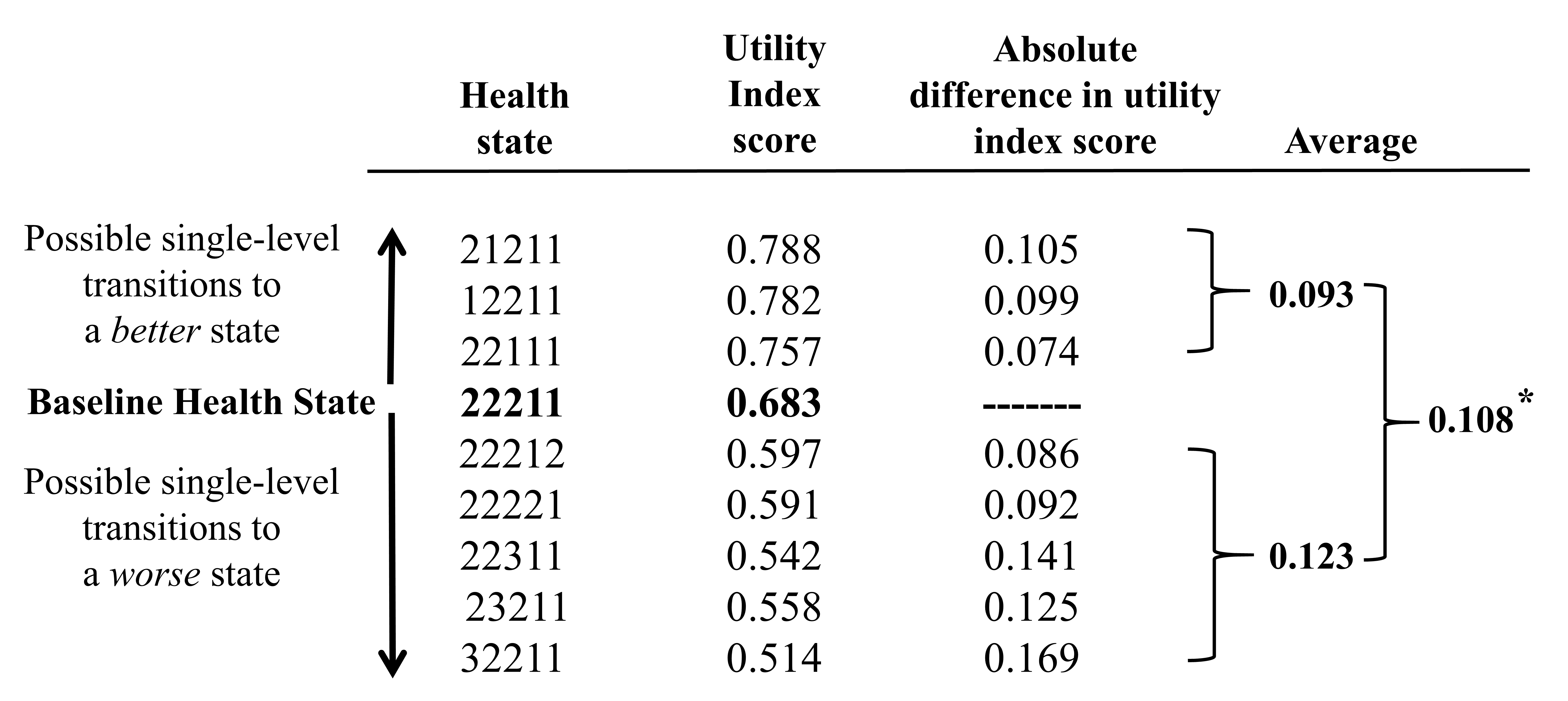


**eFigure 6.** Calculation of the instrument-defined MID estimate for the EQ-5D-3L index score on the basis of the Chinese scoring algorithm.

***Note.*** Top: V1; Under: V2.

The asterisk (*) denotes the average value of all changes, including both deterioration and improvement. Values have been rounded to three decimal places for display purposes, and thus the MlD estimate differs as a result of rounding error.


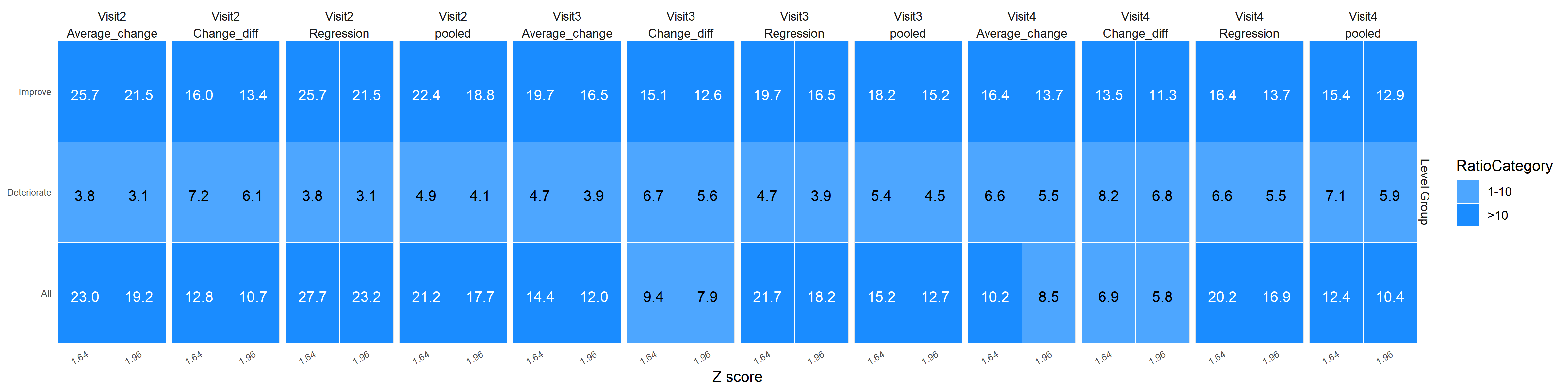


**eFigure 7.** The ratio of minimal importance differences (MID) to minimal detectable change (MDC) at the group level.

***Note.*** The blue box indicates that the group‑level MID exceeded measurement error.


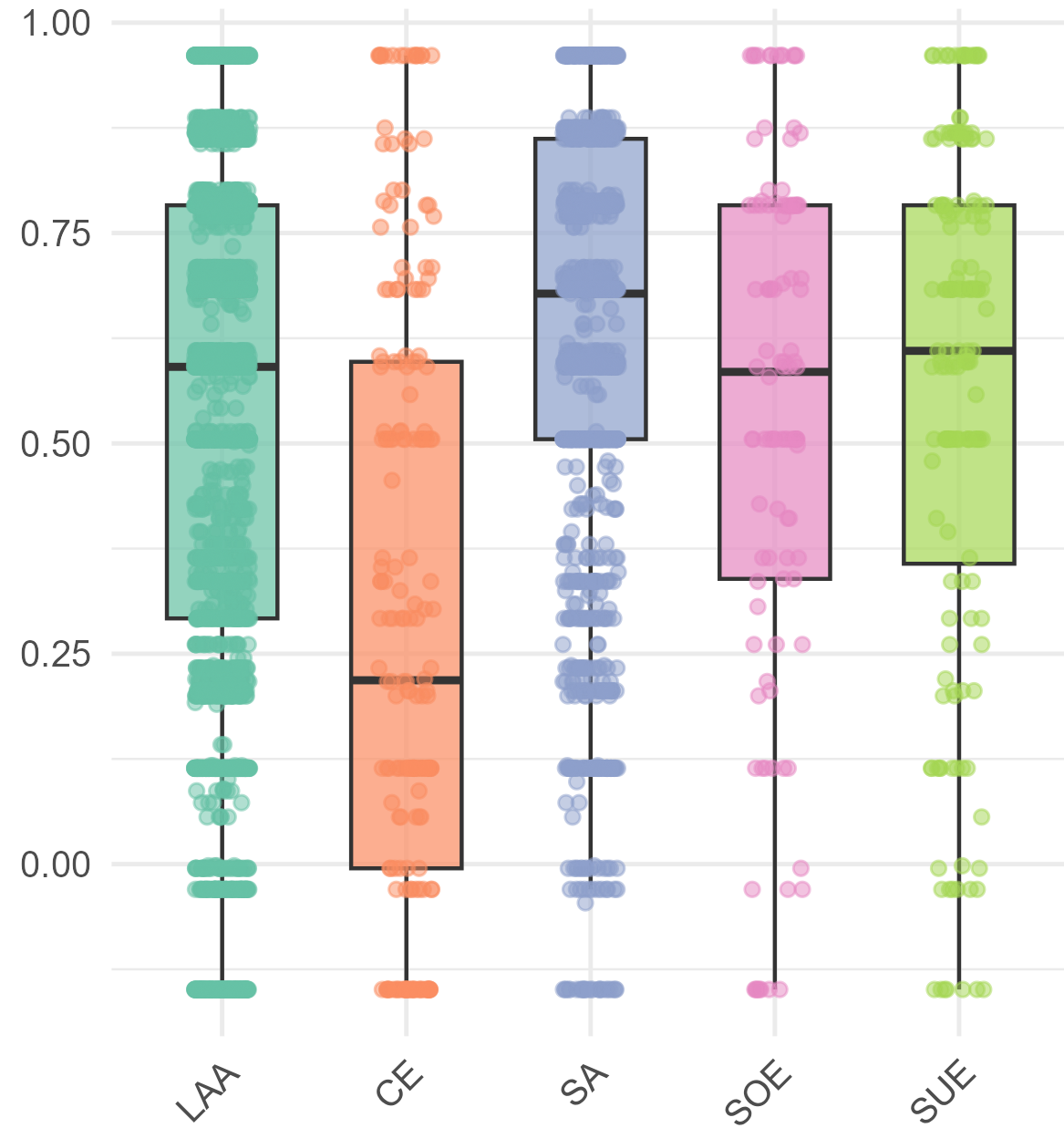

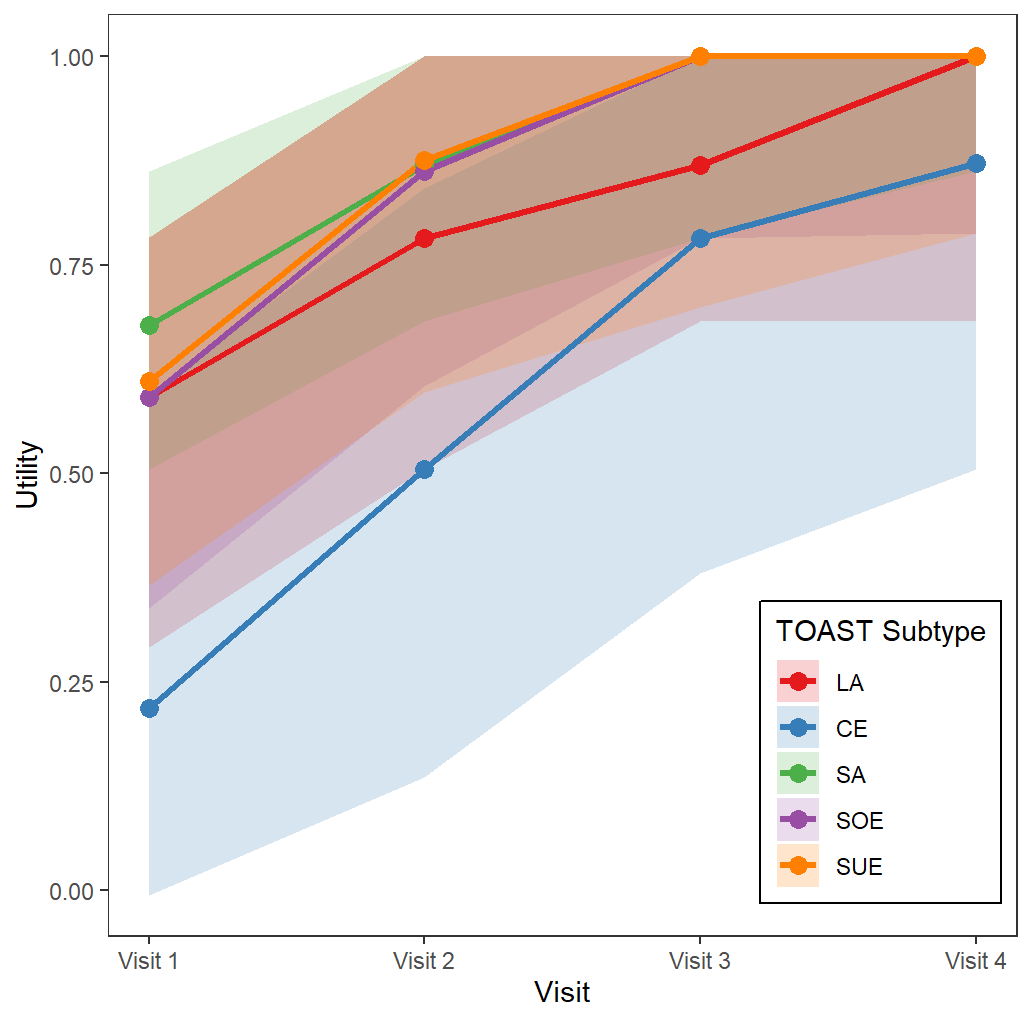


**eFigure 8.** Baseline utility distribution (left) and utility change (right) by TOAST subtype.

***Note.*** Left: Boxplot (center=median, box=IQR); Right: Trend lines (line=median, ribbon=IQR)


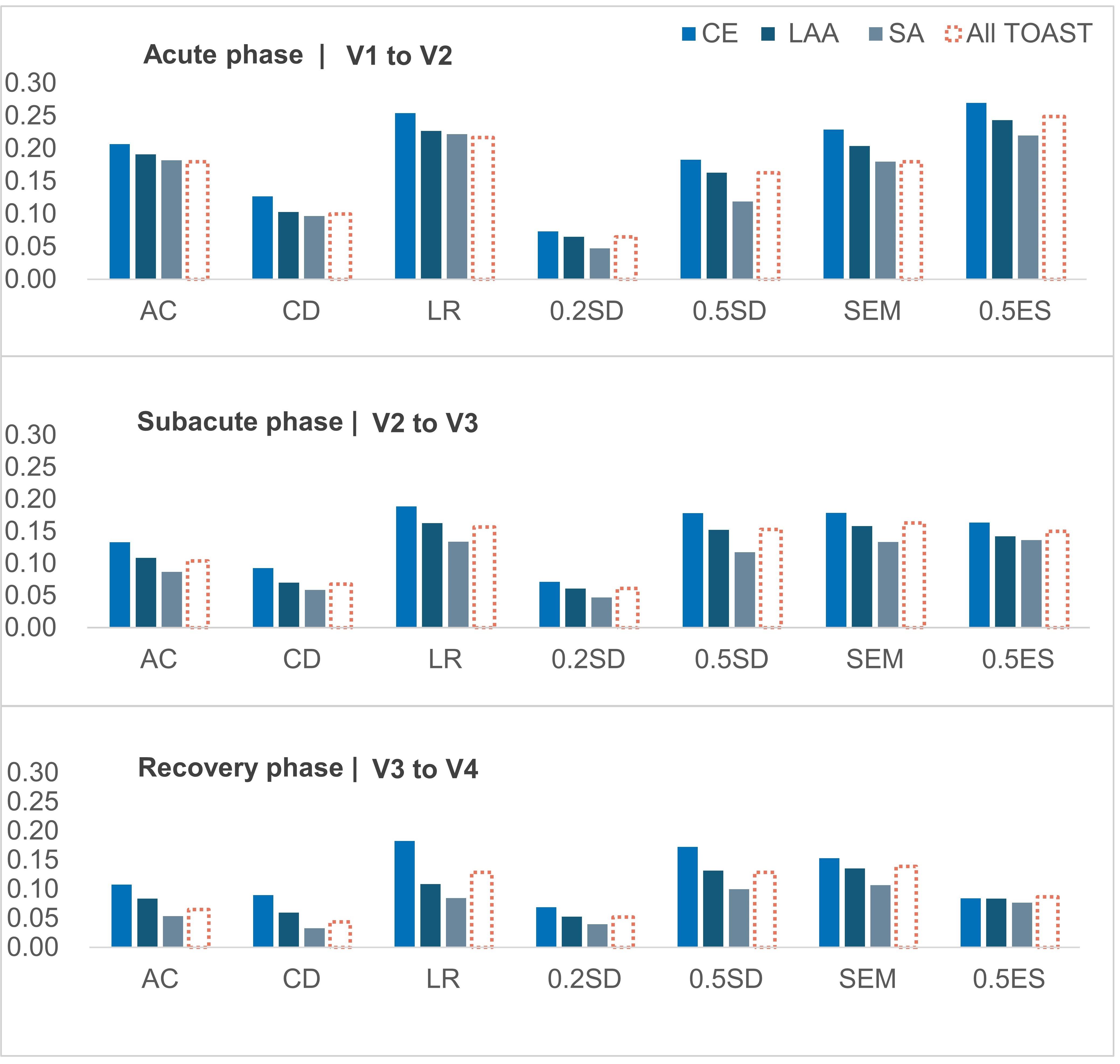


**eFigure 9.** Overall minimal importance differences (MIDs) across three TOAST subtypes for all directions of change.

Abbreviations: AC, average change; CD, change difference; ROC, receiver operating characteristic; ES, effect size; NA, not applicable; SD, standard deviation; SEM, standard error of measuremen; CE, cardioembolic infarction; LAA, large-artery atherosclerosis; SA, small-artery occlusion; t.

***Note*** Overall MIDs were derived by pooling minimal improvement and minimal deterioration, with Δ utility for deterioration multiplied by –1.

**
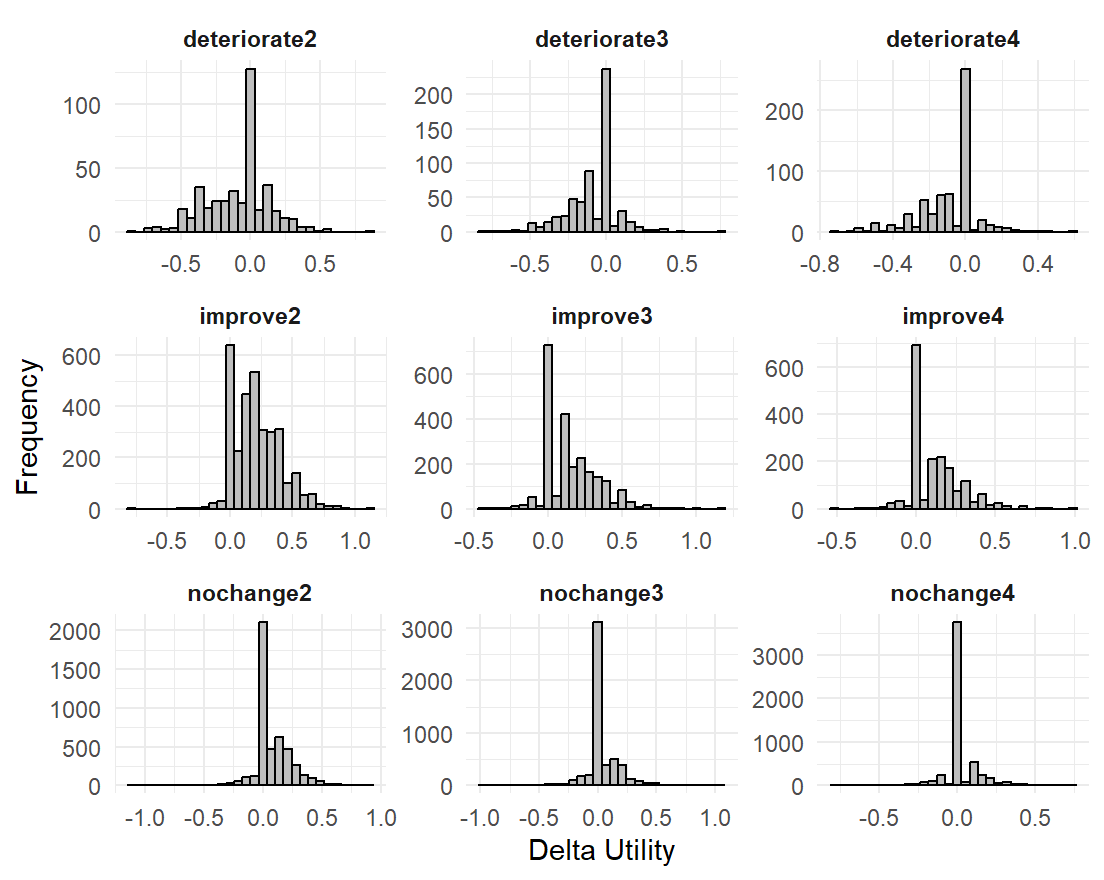
**

**eFigure 10.** Distribution of Δ utility (Δ mRS=1, -1, 0) across three phases.

***Note.*** The suffixes 2, 3, and 4 in category labels (eg, Improve2) represent changes during the acute (V1–V2), subacute (V2–V3), and chronic (V3–V4) phases, respectively.
