# Supplementary material for: Interpreting patient-reported outcomes after ischemic stroke: defining minimal important difference in EQ-5D across recovery phases
Source: Health Qual Life Outcomes. 2026 Mar 21;24:57. doi: 10.1186/s12955-026-02524-w (PMC13130762; doi:10.1186/s12955-026-02524-w)
Supplement: Supplementary file 2 — Supplementary Material 2 [file 12955_2026_2524_MOESM2_ESM.docx]

**eMethods**

R Version 4.4.3 code to accompany primary analyses

***# Note:*** The Chinese Acute Ischaemic Stroke Treatment Outcome Registry (CASTOR) database includes follow-up visits from Visit1 to Visit5. Based on the objectives and requirements of this study, data from Visit1, Visit3, Visit4, and Visit5 were utilized. In the main text, these are denoted as Visit 1, Visit 2, Visit 3, and Visit 4, respectively (with **Visit 2–4 corresponding to the original Visit3–5**).

# anchor-based----

## 1. Average change----

bootstrap_mean <- function(data, indices) {

sample_data <- data[indices]

mean(sample_data, na.rm = TRUE)}

format_ci <- function(ci) {

if (!is.null(ci$basic)) {

lower <- round(ci$basic[4], 3)

upper <- round(ci$basic[5], 3)

merged <- paste0("[", lower, ", ", upper, "]")

return(list(lower = lower, upper = upper, merged = merged))

}

list(lower = NA, upper = NA, merged = "No data")}

analyze_mid <- function(data, mrs_var, utility_var, prev_mrs_var, prev_utility_var) {

delta_mrs <- paste0("delta_", mrs_var)

delta_utility <- paste0("delta_", utility_var)

data[[delta_mrs]] <- data[[mrs_var]] - data[[prev_mrs_var]]

data[[delta_utility]] <- data[[utility_var]] - data[[prev_utility_var]]

result_df <- data.frame(

Visit = sub("mRS", "Visit", mrs_var),

Group = c("Mild worsening (ΔmRS = 1)", "Mild improvement (ΔmRS = -1)", "Mild change (|ΔmRS| = 1)"),

SampleSize = NA,

MeanDeltaUtility = NA,

CI_Lower = NA,

CI_Upper = NA,

CI_Combined = NA,

BaselineVisit = sub("mRS", "Visit", prev_mrs_var),

stringsAsFactors = FALSE)

worsening <- data[data[[delta_mrs]] == 1, ]

if (nrow(worsening) > 0) {

mean_ut <- round(mean(worsening[[delta_utility]], na.rm = TRUE), 3)

set.seed(123)

boot_res <- boot(data = worsening[[delta_utility]], statistic = bootstrap_mean, R = 5000)

ci_res <- format_ci(boot.ci(boot_res, type = "basic"))

result_df[1, ] <- c(

sub("mRS", "Visit", mrs_var),

"Mild worsening (ΔmRS = 1)",

nrow(worsening),

mean_ut,

ci_res$lower,

ci_res$upper,

ci_res$merged,

sub("mRS", "Visit", prev_mrs_var))

cat("Mild worsening (ΔmRS = 1):\nMean =", mean_ut, ", 95% CI =", ci_res$merged, "\n\n")

} else {

result_df[1, c("SampleSize", "CI_Combined")] <- c(0, "No sample")

cat("Mild worsening (ΔmRS = 1): No eligible samples\n\n")}

improvement <- data[data[[delta_mrs]] == -1, ]

if (nrow(improvement) > 0) {

mean_ut <- round(mean(improvement[[delta_utility]], na.rm = TRUE), 3)

set.seed(123)

boot_res <- boot(data = improvement[[delta_utility]], statistic = bootstrap_mean, R = 5000)

ci_res <- format_ci(boot.ci(boot_res, type = "basic"))

result_df[2, ] <- c(

sub("mRS", "Visit", mrs_var),

"Mild improvement (ΔmRS = -1)",

nrow(improvement),

mean_ut,

ci_res$lower,

ci_res$upper,

ci_res$merged,

sub("mRS", "Visit", prev_mrs_var))

cat("Mild improvement (ΔmRS = -1):\nMean =", mean_ut, ", 95% CI =", ci_res$merged, "\n\n")} else {result_df[2, c("SampleSize", "CI_Combined")] <- c(0, "No sample")

cat("Mild improvement (ΔmRS = -1): No eligible samples\n\n")}

mild_change <- data[data[[delta_mrs]] %in% c(1, -1), ]

if (nrow(mild_change) > 0) {

all_ut <- mild_change[[delta_utility]]

mean_ut <- round(mean(all_ut, na.rm = TRUE), 3)

set.seed(123)

boot_res <- boot(data = all_ut, statistic = bootstrap_mean, R = 5000)

ci_res <- format_ci(boot.ci(boot_res, type = "basic"))

result_df[3, ] <- c(

sub("mRS", "Visit", mrs_var),

"Mild change (|ΔmRS| = 1)",

nrow(mild_change),

mean_ut,

ci_res$lower,

ci_res$upper,

ci_res$merged,

sub("mRS", "Visit", prev_mrs_var))

cat("Mild change (|ΔmRS| = 1):\nMean =", mean_ut, ", 95% CI =", ci_res$merged, "\n\n")

} else {

result_df[3, c("SampleSize", "CI_Combined")] <- c(0, "No sample")

cat("Mild change (|ΔmRS| = 1): No eligible samples\n\n"

list(data = data, result = result_df)}

cat("============ Visit 3 Results ============\n")

v3_out <- analyze_mid(v3_data, "mRS3", "utility3", "mRS1", "utility1")

v3_data <- v3_out$data

cat("============ Visit 4 Results ============\n")

v4_out <- analyze_mid(v4_data, "mRS4", "utility4", "mRS3", "utility3")

v4_data <- v4_out$data

cat("============ Visit 5 Results ============\n")

v5_out <- analyze_mid(v5_data, "mRS5", "utility5", "mRS4", "utility4")

v5_data <- v5_out$data

## 2. Change difference----

compare_with_nochange <- function(data, group_type, delta_mrs_col, delta_utility_col, visit) {

nochange <- data[data[[delta_mrs_col]] == 0, , drop = FALSE]

if (group_type == "worsening") {

minichange <- data[data[[delta_mrs_col]] == 1, , drop = FALSE]

group_name <- "Minor Worsening (ΔmRS=1)"

} else if (group_type == "improvement") {

minichange <- data[data[[delta_mrs_col]] == -1, , drop = FALSE]

group_name <- "Minor Improvement (ΔmRS=-1)"

} else if (group_type == "all") {

minichange <- data[data[[delta_mrs_col]] %in% c(-1, 1), , drop = FALSE]

group_name <- "Minor Change (|ΔmRS|=1)"

} else {

stop("Invalid group type: only 'worsening'/'improvement'/'all' are supported")}

if (nrow(nochange) == 0 || nrow(minichange) == 0) {

cat(group_name, "or No Change group: Insufficient sample size (both need ≥1)\n\n")

return(data.frame(Visit = visit, Group_Name = group_name, Change_Group_Size = nrow(minichange), Change_Group_NA = sum(is.na(minichange[[delta_utility_col]])), No_Change_Size = nrow(nochange), No_Change_NA = sum(is.na(nochange[[delta_utility_col]])), Mean_Difference_CD = NA, CI_Lower = NA, CI_Upper = NA, CI_Combined = "NA-NA", Result_Note = "Insufficient sample size (both need ≥1)", stringsAsFactors = FALSE))}

mini_utility <- minichange[[delta_utility_col]]

no_utility <- nochange[[delta_utility_col]]

na_mini <- sum(is.na(mini_utility))

na_no <- sum(is.na(no_utility))

valid_mini <- sum(!is.na(mini_utility))

valid_no <- sum(!is.na(no_utility))

if (valid_mini < 3 || valid_no < 3) {

cat(group_name, "or No Change group: Insufficient valid sample size (need ≥3 non-NA values), cannot calculate confidence interval\n\n")

return(data.frame(Visit=visit,Group_Name=group_name,Change_Group_Size=nrow(minichange),Change_Group_NA=na_mini,No_Change_Size=nrow(nochange),No_Change_NA=na_no,Mean_Difference_CD=NA,CI_Lower=NA,CI_Upper=NA,CI_Combined="NA-NA",Result_Note="Insufficientvalidsamplesize(need≥3non-NAvalues)",stringsAsFactors = FALSE))}

if (na_mini / length(mini_utility) > 0.5 || na_no / length(no_utility) > 0.5) {

cat(group_name, "or No Change group: Utility value missing rate exceeds 50%, results may be unreliable\n\n")

result_note <- "Utility value missing rate exceeds 50%, results may be unreliable"

} else {

result_note <- "Normal"}

calc_mean_diff <- function(mini_data, no_data) {

mini_samp <- sample(mini_data, size = length(mini_data), replace = TRUE)

no_samp <- sample(no_data, size = length(no_data), replace = TRUE)

mean_mini_samp <- mean(mini_samp, na.rm = TRUE)

mean_no_samp <- mean(no_samp, na.rm = TRUE)

if (is.na(mean_mini_samp) || is.na(mean_no_samp)) return(NA)

return(mean_mini_samp - mean_no_samp)}

mean_mini_base <- mean(mini_utility, na.rm = TRUE)

mean_no_base <- mean(no_utility, na.rm = TRUE)

base_mean_diff <- mean_mini_base - mean_no_base

set.seed(123)

boot_results <- boot(

data = list(mini = mini_utility, no = no_utility),

statistic = function(data, indices) {calc_mean_diff(data$mini, data$no)},

R = 5000)

boot_diffs <- boot_results$t

iqr <- IQR(boot_diffs, na.rm = TRUE)

lower_bound <- quantile(boot_diffs, 0.25, na.rm = TRUE) - 1.5*iqr

upper_bound <- quantile(boot_diffs, 0.75, na.rm = TRUE) + 1.5*iqr

boot_diffs_clean <- boot_diffs[boot_diffs >= lower_bound & boot_diffs <= upper_bound]

if (length(boot_diffs_clean) >= 5000) {

ci_lower <- round(quantile(boot_diffs_clean, 0.025), 3)

ci_upper <- round(quantile(boot_diffs_clean, 0.975), 3)

ci_combined <- sprintf("[%.3f, %.3f]", ci_lower, ci_upper)

} else {

ci_lower <- NA

ci_upper <- NA

ci_combined <- "NA-NA"

result_note <- paste(result_note, "(Insufficient valid samples after filtering)", sep = " ")}

cat(group_name, "vs No Change group (CD statistic):\n")

cat(" Sample size:", group_name, length(mini_utility), "cases (NA:", na_mini, "cases); No Change group", length(no_utility), "cases (NA:", na_no, "cases)\n")

cat(" Mean difference (CD statistic):", round(base_mean_diff, 3), ", 95% confidence interval:", ci_combined, "\n\n")

return(data.frame(Visit = visit, Group_Name = group_name, Change_Group_Size = nrow(minichange), Change_Group_NA = na_mini, No_Change_Size = nrow(nochange), No_Change_NA = na_no, Mean_Difference_CD = round(base_mean_diff, 3), CI_Lower = ci_lower, CI_Upper = ci_upper, CI_Combined = ci_combined, Result_Note = result_note, stringsAsFactors = FALSE))}analyze_groups <- function(data_list, visit_list) {

if (length(data_list) != length(visit_list)) {

stop("Data list and visit list must have the same length!")}

all_results <- data.frame()

for (i in seq_along(data_list)) {

data <- data_list[[i]]

visit <- visit_list[[i]]

delta_mrs_col <- paste0("delta_mRS", visit)

delta_utility_col <- paste0("delta_utility", visit)

if (!all(c(delta_mrs_col, delta_utility_col) %in% colnames(data))) {

warning(sprintf("Visit %d: Missing required columns (%s/%s), skipping analysis", visit, delta_mrs_col, delta_utility_col))

skip_result <- data.frame(Visit=visit,Group_Name="AllGroups",Change_Group_Size=NA,Change_Group_NA=NA,No_Change_Size=NA,No_Change_NA=NA,Mean_Difference_CD=NA,CI_Lower=NA,CI_Upper=NA,CI_Combined="NA-NA",Result_Note=sprintf("Missingrequiredcolumns(%s/%s)",delta_mrs_col,delta_utility_col),stringsAsFactors=FALSE)

all_results <- rbind(all_results, skip_result)

next}

group_types <- c("worsening", "improvement", "all")

for (group_type in group_types) {

group_result <- compare_with_nochange(data=data,group_type=group_type,delta_mrs_col=delta_mrs_col,delta_utility_col=delta_utility_col,visit=visit)

all_results <- rbind(all_results, group_result)}}

return(all_results)}

## 3. Linear regression----

predict_with_ci <- function(model, new_data) {

prediction <- predict(model, newdata = new_data, interval = "confidence")

return(prediction)}

model_im3 <- lm(delta_utility3 ~ delta_mRS3, data = improve_df3)

new_data3 <- data.frame(delta_mRS3 = -1)

prediction3 <- predict(model_im3, newdata = new_data3, interval = "confidence", level = 0.95)

cat("model: delta_utility3 ~ delta_mRS3\n")

cat("regression equation: utility =", coef(model_im3)[1], "+", coef(model_im3)[2], "* delta_mRS3\n")

cat(" prediction value =", round(prediction3[1, "fit"], 3), "\n")

cat(" 95%CI = [",

round(prediction3[1,"lwr"], 3), ", ",

round(prediction3[1,"upr"], 3), "]\n\n")

model_im4 <- lm(delta_utility4 ~ delta_mRS4, data = improve_df4)

new_data4 <- data.frame(delta_mRS4 = -1)

prediction4 <- predict(model_im4, newdata = new_data4, interval = "confidence", level = 0.95)

cat("model: delta_utility4 ~ delta_mRS4\n")

cat("regression equation: utility =", coef(model_im4)[1], "+", coef(model_im4)[2], "* delta_mRS4\n")

cat(" prediction value =", round(prediction4[1, "fit"], 3), "\n")

cat(" 95%CI = [",

round(prediction4[1,"lwr"], 3), ", ",

round(prediction4[1,"upr"], 3), "]\n\n")

model_im5 <- lm(delta_utility5 ~ delta_mRS5, data = improve_df5)

new_data5 <- data.frame(delta_mRS5 = -1)

prediction5 <- predict(model_im5, newdata = new_data5, interval = "confidence", level = 0.95)

cat("model 1-year vs 3-month: delta_utility5 ~ delta_mRS5\n")

cat("regression equation: utility =", coef(model_im5)[1], "+", coef(model_im5)[2], "* delta_mRS5\n")

cat(" prediction value =", round(prediction5[1, "fit"], 3), "\n")

cat(" 95%CI = [",

round(prediction5[1,"lwr"], 3), ", ",

round(prediction5[1,"upr"], 3), "]\n")

# model: delta_utility3 ~ delta_mRS3

model_wo3 <- lm(delta_utility3 ~ delta_mRS3, data = worsen_df3)

new_data3 <- data.frame(delta_mRS3 = 1)

prediction3 <- predict(model_wo3, newdata = new_data3, interval = "confidence", level = 0.95)

cat("model: delta_utility3 ~ delta_mRS3\n")

cat("regression equation: utility =", coef(model_wo3)[1], "+", coef(model_wo3)[2], "* delta_mRS3\n")

cat(" prediction value =", round(prediction3[1, "fit"], 3), "\n")

cat(" 95%CI = [",

round(prediction3[1,"lwr"], 3), ", ",

round(prediction3[1,"upr"], 3), "]\n\n")

# model: delta_utility4 ~ delta_mRS4

model_wo4 <- lm(delta_utility4 ~ delta_mRS4, data = worsen_df4)

new_data4 <- data.frame(delta_mRS4 = 1)

prediction4 <- predict(model_wo4, newdata = new_data4, interval = "confidence", level = 0.95)

cat("model: delta_utility4 ~ delta_mRS4\n")

cat("regression equation: utility =", coef(model_wo4)[1], "+", coef(model_wo4)[2], "* delta_mRS4\n")

cat(" prediction value =", round(prediction4[1, "fit"], 3), "\n")

cat(" 95%CI = [",

round(prediction4[1,"lwr"], 3), ", ",

round(prediction4[1,"upr"], 3), "]\n\n")

# model: delta_utility5 ~ delta_mRS5

model_wo5 <- lm(delta_utility5 ~ delta_mRS5, data = worsen_df5)

new_data5 <- data.frame(delta_mRS5 = 1)

prediction5 <- predict(model_wo5, newdata = new_data5, interval = "confidence", level = 0.95)

cat("model: delta_utility5 ~ delta_mRS5\n")

cat("regression equation: utility =", coef(model_wo5)[1], "+", coef(model_wo5)[2], "* delta_mRS5\n")

cat("当 delta_mRS5 = 1 时:\n")

cat(" prediction value =", round(prediction5[1, "fit"], 3), "\n")

cat(" 95%CI = [",

round(prediction5[1,"lwr"], 3), ", ",

round(prediction5[1,"upr"], 3), "]\n")

# model: delta_utility3 ~ delta_mRS3

model_all3 <- lm(abs_delta_utility3 ~ abs_delta_mRS3, data = allchange_df3)

new_data3 <- data.frame(abs_delta_mRS3 = 1)

prediction3 <- predict(model_all3, newdata = new_data3, interval = "confidence", level = 0.95)

cat("model: delta_utility3 ~ delta_mRS3\n")

cat("regression equation: utility =", coef(model_all3)[1], "+", coef(model_all3)[2], "* delta_mRS3\n")

cat(" prediction value =", round(prediction3[1, "fit"], 3), "\n")

cat(" 95%CI = [",

round(prediction3[1,"lwr"], 3), ", ",

round(prediction3[1,"upr"], 3), "]\n\n")

# model: delta_utility4 ~ delta_mRS4

model_all4 <- lm(abs_delta_utility4 ~ abs_delta_mRS4, data = allchange_df4)

new_data4 <- data.frame(abs_delta_mRS4 = 1)

prediction4 <- predict(model_all4, newdata = new_data4, interval = "confidence", level = 0.95)

cat("model: delta_utility4 ~ delta_mRS4\n")

cat("regression equation: utility =", coef(model_all4)[1], "+", coef(model_all4)[2], "* delta_mRS4\n")

cat(" prediction value =", round(prediction4[1, "fit"], 3), "\n")

cat(" 95%CI = [",

round(prediction4[1,"lwr"], 3), ", ",

round(prediction4[1,"upr"], 3), "]\n\n")

# model: delta_utility5 ~ delta_mRS5

model_all5 <- lm(abs_delta_utility5 ~ abs_delta_mRS5, data = allchange_df5)

new_data5 <- data.frame(abs_delta_mRS5 = 1)

prediction5 <- predict(model_all5, newdata = new_data5, interval = "confidence", level = 0.95)

cat("model: delta_utility5 ~ delta_mRS5\n")

cat("regression equation: utility =", coef(model_all5)[1], "+", coef(model_all5)[2], "* delta_mRS5\n")

cat(" prediction value =", round(prediction5[1, "fit"], 3), "\n")

cat(" 95%CI = [",

round(prediction5[1,"lwr"], 3), ", ",

round(prediction5[1,"upr"], 3), "]\n")

## 4. Receiver operating characteristic----

perform_roc_analysis <- function(data, utility_col, visit_name, n_boot = 5000) {

roc_obj <- roc(data$gold_label, data[[utility_col]],

direction = ">",#improve <

auc = TRUE, ci = TRUE)

best_coords <- coords(roc_obj, "best", ret = c("threshold", "specificity", "sensitivity"),

best.method = "youden", transpose = TRUE)

set.seed(123)

boot_func <- function(data, indices, utility_col) {

d <- data[indices, ]

roc_boot <- roc(d$gold_label, d[[utility_col]], direction = ">", quiet = TRUE)

coords_boot <- coords(roc_boot, "best", ret = "threshold",

best.method = "youden", transpose = TRUE)

return(coords_boot)}

boot_results <- boot(data, boot_func, R = n_boot, utility_col = utility_col)

boot_ci <- boot.ci(boot_results, type = "perc")

results <- data.frame(

Visit = visit_name,

AUC = roc_obj$auc,

AUC_CI_lower = roc_obj$ci[1],

AUC_CI_upper = roc_obj$ci[3],

Best_Cutoff = best_coords["threshold"],

Cutoff_CI_lower = ifelse(!is.null(boot_ci$percent), boot_ci$percent[4], NA),

Cutoff_CI_upper = ifelse(!is.null(boot_ci$percent), boot_ci$percent[5], NA),

Sensitivity = best_coords["sensitivity"],

Specificity = best_coords["specificity"],

Youden_Index = best_coords["sensitivity"] + best_coords["specificity"] - 1

)

return(list(roc_obj = roc_obj, results = results))

}

perform_roc_analysis <- function(data, utility_col, visit_name, n_boot = 5000) {

roc_obj <- roc(data$gold_label, data[[utility_col]],

direction = "<", # improve

auc = TRUE, ci = TRUE)

best_coords <- coords(roc_obj, "best", ret = c("threshold", "specificity", "sensitivity"),

best.method = "youden", transpose = TRUE)

set.seed(123)

boot_func <- function(data, indices, utility_col) {

d <- data[indices, ]

tryCatch({

roc_boot <- roc(d$gold_label, d[[utility_col]], direction = "<", quiet = TRUE)

coords_boot <- coords(roc_boot, "best", ret = "threshold",

best.method = "youden", transpose = TRUE)

if (length(coords_boot) == 0 || is.na(coords_boot)) {

return(NA)

} else {

return(as.numeric(coords_boot))

}

}, error = function(e) {

return(NA)

})

}

tryCatch({

boot_results <- boot(data, boot_func, R = n_boot, utility_col = utility_col)

valid_results <- boot_results$t[!is.na(boot_results$t)]

if (length(valid_results) > 0) {

boot_ci <- quantile(valid_results, c(0.025, 0.975), na.rm = TRUE)

} else {

boot_ci <- c(NA, NA)}

}, error = function(e) {

boot_ci <- c(NA, NA)

boot_results <- NULL})

results <- data.frame(

Visit=visit_name,AUC=roc_obj$auc,AUC_CI_lower=roc_obj$ci[1],AUC_CI_upper=roc_obj$ci[3],Best_Cutoff=best_coords["threshold"],Cutoff_CI_lower=ifelse(exists("boot_ci"),boot_ci[1],NA),Cutoff_CI_upper=ifelse(exists("boot_ci"),boot_ci[2],NA),Sensitivity=best_coords["sensitivity"],Specificity=best_coords["specificity"],Youden_Index = best_coords["sensitivity"] + best_coords["specificity"] - 1)

return(list(roc_obj = roc_obj, results = results))}

###worsen----

worsen3 <- v3_data %>%

filter(delta_mRS3 %in% c(0, 1)) %>%

dplyr::select(PT, delta_mRS3, delta_utility3)%>%

mutate(

gold_label = ifelse(delta_mRS3 == 1, 1, 0)

)

worsen4 <- v4_data %>%

filter(delta_mRS4 %in% c(0, 1)) %>%

dplyr::select(PT, delta_mRS4, delta_utility4)%>%

mutate(

gold_label = ifelse(delta_mRS4 == 1, 1, 0)

)

worsen5 <- v5_data %>%

filter(delta_mRS5 %in% c(0, 1)) %>%

dplyr::select(PT, delta_mRS5, delta_utility5)%>%

mutate(

gold_label = ifelse(delta_mRS5 == 1, 1, 0)

)

roc_worsen3 <- perform_roc_analysis(worsen3, "delta_utility3", "Visit 2")

roc_worsen4 <- perform_roc_analysis(worsen4, "delta_utility4", "Visit 3")

roc_worsen5 <- perform_roc_analysis(worsen5, "delta_utility5", "Visit 4")

###improve----

improve3 <- v3_data %>%

filter(delta_mRS3 %in% c(0, -1)) %>%

dplyr::select(PT, delta_mRS3, delta_utility3)%>%

mutate(

gold_label = ifelse(delta_mRS3 == -1, 1, 0)

)

improve4 <- v4_data %>%

filter(delta_mRS4 %in% c(0, -1)) %>%

dplyr::select(PT, delta_mRS4, delta_utility4)%>%

mutate(

gold_label = ifelse(delta_mRS4 == -1, 1, 0)

)

improve5 <- v5_data %>%

filter(delta_mRS5 %in% c(0, -1)) %>%

dplyr::select(PT, delta_mRS5, delta_utility5)%>%

mutate(

gold_label = ifelse(delta_mRS5 == -1, 1, 0)

)

roc_improve3 <- perform_roc_analysis(improve3, "delta_utility3", "Visit 2")

roc_improve4 <- perform_roc_analysis(improve4, "delta_utility4", "Visit 3")

roc_improve5 <- perform_roc_analysis(improve5, "delta_utility5", "Visit 4")

###overall----

all3 <- bind_rows(

improve3,

worsen3 %>% mutate(delta_utility3 = delta_utility3 * -1))%>%

distinct(PT, .keep_all = TRUE)

all4 <- bind_rows(

improve4,

worsen4 %>% mutate(delta_utility4 = delta_utility4 * -1))%>%

distinct(PT, .keep_all = TRUE)

all5 <- bind_rows(

improve5,

worsen5 %>% mutate(delta_utility5 = delta_utility5 * -1))%>%

distinct(PT, .keep_all = TRUE)

roc_all3 <- perform_roc_analysis(all3, "delta_utility3", "Visit 2")

roc_all4 <- perform_roc_analysis(all4, "delta_utility4", "Visit 3")

roc_all5 <- perform_roc_analysis(all5, "delta_utility5", "Visit 4")

all_results <- rbind( roc_improve3$results,roc_improve4$results,roc_improve5$results,roc_worsen3$results,roc_worsen4$results,roc_worsen5$results,roc_all3$results,roc_all4$results,roc_all5$results)

all_results <- rbind()

all_results <- rbind(

# distribution-based----

###select dataset (delta_mRS <= 0)

improve3 <- v3_data %>%

filter(delta_mRS3 <= 0) %>%

dplyr::select(PT, delta_mRS3, delta_utility3, utility3,mRS3)

improve4 <- v4_data %>%

filter(delta_mRS4 <= 0) %>%

dplyr::select(PT, delta_mRS4, delta_utility4, utility3, utility4,mRS4)

improve5 <- v5_data %>%

filter(delta_mRS5 <= 0) %>%

dplyr::select(PT, delta_mRS5, delta_utility5, utility4, utility5,mRS5)

### select dataset (delta_mRS >= 0)

worsen3 <- v3_data %>%

filter(delta_mRS3 >=0 ) %>%

dplyr::select(PT, delta_mRS3, delta_utility3, utility3,mRS3)

worsen4 <- v4_data %>%

filter(delta_mRS4 >=0 ) %>%

dplyr::select(PT, delta_mRS4, delta_utility4, utility3, utility4,mRS4)

worsen5 <- v5_data %>%

filter(delta_mRS5 >=0) %>%

dplyr::select(PT, delta_mRS5, delta_utility5, utility4, utility5,mRS5)

## 1. 0.2 × SD----

baseline_sd_3all <- 0.2*sd(v1_data$utility1, na.rm = TRUE)

baseline_sd_3all

baseline_sd <- sd(v1_data$utility1, na.rm = TRUE)

MID_SD <- 0.2 * baseline_sd

cat("3all MID_SD =", round(MID_SD, 3))

baseline_sd <- sd(v3_data$utility3, na.rm = TRUE)

MID_SD <- 0.2 * baseline_sd

cat("4all MID_SD =", round(MID_SD, 3))

baseline_sd <- sd(v4_data$utility4, na.rm = TRUE)

MID_SD <- 0.2 * baseline_sd

cat("5all MID_SD =", round(MID_SD, 3))

baseline_sd <- sd(improve1$utility1, na.rm = TRUE)

MID_SD <- 0.2 * baseline_sd

cat("3+MID_SD =", round(MID_SD, 3))

baseline_sd <- sd(improve3$utility3, na.rm = TRUE)

MID_SD <- 0.2 * baseline_sd

cat("4+MID_SD =", round(MID_SD, 3))

baseline_sd <- sd(improve4$utility4, na.rm = TRUE)

MID_SD <- 0.2 * baseline_sd

cat("5+MID_SD =", round(MID_SD, 3))

baseline_sd <- sd(worsen1$utility1, na.rm = TRUE)

MID_SD <- 0.2 * baseline_sd

cat("3-MID_SD =", round(MID_SD, 3))

baseline_sd <- sd(worsen3$utility3, na.rm = TRUE)

MID_SD <- 0.2 * baseline_sd

cat("4-MID_SD =", round(MID_SD, 3))

baseline_sd <- sd(worsen4$utility4, na.rm = TRUE)

MID_SD <- 0.2 * baseline_sd

cat("5-MID_SD =", round(MID_SD, 3))

## 2. 0.5 × SD----

baseline_sd_3all <- 0.5*sd(v1_data$utility1, na.rm = TRUE)

baseline_sd_3all

baseline_sd <- sd(v1_data$utility1, na.rm = TRUE)

MID_SD <- 0.5 * baseline_sd

cat("3all MID_SD =", round(MID_SD, 3))

baseline_sd <- sd(v3_data$utility3, na.rm = TRUE)

MID_SD <- 0.5 * baseline_sd

cat("4all MID_SD =", round(MID_SD, 3))

baseline_sd <- sd(v4_data$utility4, na.rm = TRUE)

MID_SD <- 0.5 * baseline_sd

cat("5all MID_SD =", round(MID_SD, 3))

baseline_sd <- sd(improve1$utility1, na.rm = TRUE)

MID_SD <- 0.5 * baseline_sd

cat("3+MID_SD =", round(MID_SD, 3))

baseline_sd <- sd(improve3$utility3, na.rm = TRUE)

MID_SD <- 0.5 * baseline_sd

cat("4+MID_SD =", round(MID_SD, 3))

baseline_sd <- sd(improve4$utility4, na.rm = TRUE)

MID_SD <- 0.5 * baseline_sd

cat("5+MID_SD =", round(MID_SD, 3))

baseline_sd <- sd(worsen1$utility1, na.rm = TRUE)

MID_SD <- 0.5 * baseline_sd

cat("3-MID_SD =", round(MID_SD, 3))

baseline_sd <- sd(worsen3$utility3, na.rm = TRUE)

MID_SD <- 0.5 * baseline_sd

cat("4-MID_SD =", round(MID_SD, 3))

baseline_sd <- sd(worsen4$utility4, na.rm = TRUE)

MID_SD <- 0.5 * baseline_sd

cat("5-MID_SD =", round(MID_SD, 3))

## 3. 0.5 × ES----

###all

delta_mean3all <- mean(v3_data$utility3)-mean(v1_data$utility1)

delta_sd <- sd(v1_data$utility1, na.rm = TRUE)

es <- delta_mean3all / delta_sd # Cohen's d

MID_0.5ES <- 0.5 * es

cat("V3 MID_0.5ES =", round(MID_0.5ES, 3))

delta_mean4all <- mean(v4_data$utility4)-mean(v3_data$utility3)

delta_sd <- sd(v3_data$utility3, na.rm = TRUE)

es <- delta_mean4all / delta_sd # Cohen's d

MID_0.5ES <- 0.5 * es

cat("V4 MID_0.5ES =", round(MID_0.5ES, 3))

delta_mean5all <- mean(v5_data$utility5)-mean(v4_data$utility4)

delta_sd <- sd(v4_data$utility4, na.rm = TRUE)

es <- delta_mean5all / delta_sd # Cohen's d

MID_0.5ES <- 0.5 * es

cat("V5 MID_0.5ES =", round(MID_0.5ES, 3))

###improve

delta_mean3im <- mean(improve3$utility3)-mean(improve1$utility1)

delta_sd <- sd(improve1$utility1, na.rm = TRUE)

es <- delta_mean / delta_sd # Cohen's d

MID_0.5ES <- 0.5 * es

cat("V3 MID_0.5ES =", round(MID_0.5ES, 3))

delta_mean4im <- mean(improve4$utility4)-mean(improve3$utility3)

delta_sd <- sd(improve3$utility3, na.rm = TRUE)

es <- delta_mean / delta_sd # Cohen's d

MID_0.5ES <- 0.5 * es

cat("V4 MID_0.5ES =", round(MID_0.5ES, 3))

delta_mean5im <- mean(improve5$utility5)-mean(improve4$utility4)

delta_sd <- sd(improve4$utility4, na.rm = TRUE)

es <- delta_mean / delta_sd # Cohen's d

MID_0.5ES <- 0.5 * es

cat("V5 MID_0.5ES =", round(MID_0.5ES, 3))

###worsen

delta_mean3wo <- mean(worsen3$utility3)-mean(worsen1$utility1)

delta_sd <- sd(worsen1$utility1, na.rm = TRUE)

es <- delta_mean3wo / delta_sd # Cohen's d

MID_0.5ES <- 0.5 * es

cat("V3 MID_0.5ES =", round(MID_0.5ES, 3))

delta_mean4wo <- mean(worsen4$utility4)-mean(worsen3$utility3)

delta_sd <- sd(worsen3$utility3, na.rm = TRUE)

es <- delta_mean4wo / delta_sd # Cohen's d

MID_0.5ES <- 0.5 * es

cat("V4 MID_0.5ES =", round(MID_0.5ES, 3))

delta_mean5wo <- mean(worsen5$utility5)-mean(worsen4$utility4)

delta_sd <- sd(worsen4$utility4, na.rm = TRUE)

es <- delta_mean5wo / delta_sd # Cohen's d

MID_0.5ES <- 0.5 * es

cat("V5 MID_0.5ES =", round(MID_0.5ES, 3))

## 4. SEM（----

###all

baseline_sd_utility <- sd(v1_data$utility1, na.rm = TRUE)

MID_SEM3all <- baseline_sd_utility * sqrt(1 - 0.61)

baseline_sd_utility <- sd(v3_data$utility3, na.rm = TRUE)

MID_SEM4all <- baseline_sd_utility * sqrt(1 - 0.70)

baseline_sd_utility <- sd(v4_data$utility4, na.rm = TRUE)

MID_SEM5all <- baseline_sd_utility * sqrt(1 - 0.73)

cat("MID_SEM3all =", round(MID_SEM3all, 3), " | MID_SEM4all =", round(MID_SEM4all, 3), " | MID_SEM5all =", round(MID_SEM5all, 3), "\n")

###improve

baseline_sd_utility <- sd(improve1$utility1, na.rm = TRUE)

MID_SEM3im <- baseline_sd_utility * sqrt(1 - 0.61)

baseline_sd_utility <- sd(improve3$utility3, na.rm = TRUE)

MID_SEM4im <- baseline_sd_utility * sqrt(1 - 0.70)

baseline_sd_utility <- sd(improve4$utility4, na.rm = TRUE)

MID_SEM5im <- baseline_sd_utility * sqrt(1 - 0.73)

cat("MID_SEM3im =", round(MID_SEM3im, 3), " | MID_SEM4im =", round(MID_SEM4im, 3), " | MID_SEM5im =", round(MID_SEM5im, 3), "\n")

###worsen

baseline_sd_utility <- sd(worsen1$utility1, na.rm = TRUE)

MID_SEM3wo <- baseline_sd_utility * sqrt(1 - 0.61)

baseline_sd_utility <- sd(worsen3$utility3, na.rm = TRUE)

MID_SEM4wo <- baseline_sd_utility * sqrt(1 - 0.70)

baseline_sd_utility <- sd(worsen4$utility4, na.rm = TRUE)

MID_SEM5wo <- baseline_sd_utility * sqrt(1 - 0.73)

cat("MID_SEM3wo =", round(MID_SEM3wo, 3), " | MID_SEM4wo =", round(MID_SEM4wo, 3), " | MID_SEM5wo =", round(MID_SEM5wo, 3), "\n")

# instrument-defined----

v1_data$state1 <- apply(v1_data[, c("EQmob1", "EQsc1", "EQact1", "EQpain1", "EQanxie1")], 1, function(row) paste(row, collapse = ""))

v3_data$state3 <- apply(v3_data[, c("EQmob3", "EQsc3", "EQact3", "EQpain3", "EQanxie3")], 1, function(row) paste(row, collapse = ""))

v4_data$state4 <- apply(v4_data[, c("EQmob4", "EQsc4", "EQact4", "EQpain4", "EQanxie4")], 1, function(row) paste(row, collapse = ""))

baseline_state3_distribution <- v3_data %>%

count(state3, sort = TRUE) %>%

rename(frequency = n)

print(baseline_state3_distribution)

baseline_state4_distribution <- v4_data %>%

count(state4, sort = TRUE) %>%

rename(frequency = n)

print(baseline_state4_distribution)

baseline_utility <- v3_data$utility3[v3_data$state3 == "22211"]

increase_changes <- numeric(5)

increase_states <- character(5)

for(i in 1:5) {

state_vec <- strsplit("22211", "")[[1]]

state_vec[i] <- as.character(as.numeric(state_vec[i]) + 1)

new_state <- paste(state_vec, collapse = "")

increase_states[i] <- new_state

new_utility <- v3_data$utility3[v3_data$state3 == new_state]

if(length(new_utility) > 0) {

increase_utilities[i] <- new_utility

increase_changes[i] <- new_utility - baseline_utility

} else {

warning(paste("cann’t find state3 ", new_state, "record"))

increase_utilities[i] <- NA

increase_changes[i] <- NA}}

mean_increase <- mean(increase_changes, na.rm = TRUE)

cat("result：\n")

for(i in 1:5) {

if(!is.na(increase_changes[i])) {

cat(paste("state3:", increase_states[i],

"utility3:", round(increase_utilities[i], 4),

"变化:", round(increase_changes[i], 4), "\n"))}}

cat(paste("change for improve:", round(mean_increase, 4), "\n\n"))

decrease_changes <- numeric(5)

decrease_utilities <- numeric(5)

decrease_states <- character(5)

valid_count <- 0

for(i in 1:5) {

state_vec <- strsplit("22211", "")[[1]]

if(as.numeric(state_vec[i]) > 1) {

state_vec[i] <- as.character(as.numeric(state_vec[i]) - 1)

new_state <- paste(state_vec, collapse = "")

decrease_states[i] <- new_state

new_utility <- v3_data$utility3[v3_data$state3 == new_state]

if(length(new_utility) > 0) {

decrease_utilities[i] <- new_utility

decrease_changes[i] <- new_utility - baseline_utility

valid_count <- valid_count + 1

} else {

warning(paste("cann’y find state3为", new_state, "reocrd"))

decrease_utilities[i] <- NA

decrease_changes[i] <- NA

}

} else {

decrease_states[i] <- NA

decrease_utilities[i] <- NA

decrease_changes[i] <- NA

}

}

mean_decrease <- mean(decrease_changes, na.rm = TRUE)

cat("result 2：\n")

for(i in 1:5) {

if(!is.na(decrease_changes[i])) {

cat(paste("state3:", decrease_states[i],

"utility3:", round(decrease_utilities[i], 4),

"change:", round(decrease_changes[i], 4), "\n"))}}

cat(paste("change for worse:", round(mean_decrease, 4), "\n\n"))

final_result <- (abs(mean_increase) + abs(mean_decrease)) / 2

cat("result 3：\n")

cat(paste("result:", round(final_result, 4), "\n")) "utility3:", round(decrease_utilities[i], 4),

"change:", round(decrease_changes[i], 4), "\n"))}}

cat(paste("change for worse:", round(mean_decrease, 4), "\n\n"))

final_result <- (abs(mean_increase) + abs(mean_decrease)) / 2

cat("result：\n")

cat(paste("result:", round(final_result, 4), "\n"))
